# Identification of biogeographically informative microssatelite markers for Brazilian *Cannabis sativa* samples: a machine learning approach for forensic origin prediction

**DOI:** 10.1007/s00414-025-03716-7

**Published:** 2026-02-06

**Authors:** Cássio Augusto Rodrigues Bettim, Lucas de Oliveira Pereira Ribeiro, Oscar Victor Cardenas Alegría, Eduardo Filipe Avila Silva, Franciele Maboni Siqueira, Flávio Anastácio de Oliveira Camargo, Clarice Sampaio Alho, Márcio Dorn

**Affiliations:** 1 Laboratory of Structural Bioinformatics and Computational Biology, Federal University of Rio Grande do Sul, Porto Alegre, RS Brazil; 2 National Institute of Forensic Science and Technology, Porto Alegre, RS Brazil; 3 Institute of Informatics, Federal University of Rio Grande do Sul, Porto Alegre, RS Brazil; 4 Center for Biotechnology, Federal University of Rio Grande do Sul, Porto Alegre, RS Brazil; 5 Institute of Biological Sciences, Federal University of Pará, Belém, PA Brazil; 6 Pathology Post Graduation Program, Health Sciences Federal University of Porto Alegre, Porto Alegre, RS Brazil; 7 Faculty of Agronomy, Federal University of Rio Grande do Sul, Porto Alegre, RS Brazil

**Keywords:** *Cannabis sativa*, Simple sequence repeats, Machine learning, Feature selection, Origin tracking

## Abstract

In Brazil, *Cannabis sativa* is the most commonly trafficked illicit drug, with supply chains originating from both domestic and international sources, often involving sophisticated trafficking methods. In response to the Brazilian Federal Police (BFP) eradication efforts targeting large-scale plantations, alternative trafficking strategies have emerged, such as dispatching *C. sativa* seeds via postal services for indoor cultivation. In contemporary forensic genetics, microsatellites remain the gold standard for identification and have gained recognition as valuable tracking tools in investigative contexts. However, currently available microsatellite panels are not optimally informative for Brazilian samples. This study aimed to identify and evaluate highly informative microsatellites for origin tracking, using genomic data from 38 *C. sativa* samples sourced from Paraguay (PG), Colombia (CO), the Marijuana Polygon (MP) region, and a foreign group (FG) of commercial strains that constitute a substantial portion of national trafficking. Various combinations of Machine Learning (ML) Feature Selection (FS) techniques and classification models were used. To this end, diverse metrics for evaluating the performance of classification models were considered alongside the execution of principal coordinate analyzes (PCoA) and hierarchical clustering. Generally, Support Vector Classifiers (SVC) combined with different FS strategies demonstrated superior performance in predicting sample origin, achieving perfect classification of all samples using only 9 SSRs. These markers also effectively differentiated the four groups of seizures based on their biogeographic origin in PCoA and their hierarchical clustering. This study successfully identified informative SSR markers for distinguishing *C. sativa* samples associated with significant trafficking routes. Further research involving new and larger sample groups can be important to validate their application in practical investigative settings.

**Supplementary Information:**

The online version contains supplementary material available at 10.1007/s00414-025-03716-7.

## Introduction

*Cannabis sativa L.* is a dicotyledonous plant belonging to the Cannabaceae family. It is considered a socially and economically important crop, as it is increasingly cultivated and used in medicinal and recreational activities. This plant can be divided into the subspecies or varieties indica, sativa, and ruderalis, designations based on morphological characteristics (i.e., C. indica, shorter with a woody stem and *C. sativa*, taller with a fibrous stem) [19]. It can also be legally divided between hemp varieties and recreational drugs, popularly known as marijuana or weed, based on their chemical composition. It has a complex chemical profile with more than 500 constituents, including more than 100 identified phytocannabinoids, the most relevant being delta-9-tetrahydrocannabinol (THC) and cannabidiol (CBD) [32, 34].

In Brazil, the cannabis-type drug remains the most widely consumed illicit drug [28]. Approximately 30% of the country’s illegal market is supplied by cultivation on the national territory originating from the marijuana polygon, a region made up of 13 cities in the states of Pernambuco and Bahia in the northeast and the middle and sub-middle hinterland regions of the São Francisco river, which supplies the North and Northeast regions of the country. Meanwhile, the remaining 70% is mainly supplied by international trafficking coming from cultivation areas spread across the Brazil-Paraguay border, especially Mato Grosso do Sul state, which supplies the South, South-West and Central-West regions [15, 36]. In addition to these two known routes, due to dedicated efforts of the Brazilian Federal Police (BFP) in eradicating large-scale plantations, alternative forms of trafficking, such as sending *C. sativa* seeds by post logistics, have emerged in recent years [33, 40]. The seeds are imported from neighboring countries such as Uruguay. However, they are mainly from the European continent, an important international supplier, and are intended for indoor cultivation on a smaller scale, which is more difficult for police forces to locate.

Different forensic methodologies for differentiating *C. sativa* samples about their biogeographical origin have been suggested in recent years and using different types of techniques, such as DNA barcoding, amplification of genetic markers in hotspot regions such as SNPs and microsatellites, and multi-element concentration in plants and soil [6, 11, 15, 30, 35]. Although some of these methodologies have been successful in individualizing and tracking the origin of the plant’s cultivation, some of their limitations include: 1) the panels of genetic variants used in these studies were developed from foreign samples - in most cases, cultivated in Europe or North America - and do not present the most informative loci for identifying and classifying *C. sativa* that are present in the Brazilian illegal market, requiring a more significant number of markers to be needed for their differentiation, thus making the process more costly and demanding greater labor effort; 2) certain types of materials, such as seeds, young plants or more degraded samples are not suitable for chemical analysis [30, 35].

Microsatellites, also known as short tandem repeats (STR) or simple sequence repeats (SSR), serve as the cornerstone of contemporary forensic genetics, recognized as the gold standard for human identification [12, 22]. Their utility extends beyond forensics, as they are also widely employed in studies of genetic diversity and population structure in plants [42]. These markers are ubiquitously distributed across prokaryotic and eukaryotic genomes, with a particular prevalence within eukaryote euchromatin, encompassing both coding and non-coding regions. Building upon the established applications of microsatellites, recent research has demonstrated the efficacy of Machine Learning (ML) strategies in predicting various phenotypes and complex traits from multi-omics data across a diverse range of plant species. Specifically, techniques such as feature selection (FS) and the application of supervised classification models have been successfully utilized in soybeans, rice, coffee, grapevines, corn and Arabidopsis thaliana [1, 16, 20, 26, 41, 43, 45]. However, despite these advances, there is a notable gap in applying such methodologies to data derived from *C. sativa* for origin tracking. While recent studies have successfully applied ML and statistical methods to *Cannabis sativa* STR data [8, 9], these works primarily focused on establishing associations between genetic markers and chemical profiles (e.g., THC levels). To our knowledge, no study to date has used microsatellite data and ML specifically to infer the geographical provenance of *C. sativa* samples associated with trafficking routes in Latin America.

Microsatellite markers are powerful tools for elucidating population structure. Consequently, this study used these markers to determine the geographical origin of *Cannabis sativa* genomes associated with Brazilian narcotraffic routes. Specifically, we explore a range of supervised learning strategies, feature selection (FS) methods, and established classification models. We evaluated the efficacy of various combinations of these techniques to achieve a robust and accurate origin determination. This approach allowed for a comprehensive assessment of the potential of SSR markers and ML to trace the provenance of *C. sativa* samples in the context of the distribution of illicit drugs.

## Materials and methods

### Samples

This study investigated the chemical composition of 38 *C. sativa* samples provided by the Brazilian Federal Police to determine potential variations based on the culture origin. The samples were categorized into four distinct groups: Paraguay (PG), Colombia (CO), Marijuana Polygon (MP), and a Foreign Group (FG) of commercial strains with unspecified origins. PG (n=8) and CO (n=4) samples were derived from seeds extracted from compressed cannabis bricks - comprising dried leaves, flowers, and stems - seized during postal sorting operations between 2019 and 2022. Based on the analysis of imported materials, the investigation extended to domestically sourced samples. Specifically, MP samples (n=12) comprised leaf plant material confiscated during on-site BFP operations from 2015 to 2019. Complementing these domestic samples, the FG (n=14) comprised leaf plant material cultivated from seeds of various foreign commercial strains. These seeds were taken from postal services in 2017 and 2019 and subsequently cultivated by BFP for confirmation tests. This comprehensive sampling strategy allowed for a comparative assessment of *C. sativa* composition in diverse geographical and cultivation contexts. Furthermore, the collection of samples from distinct seizures over varying time periods (2015–2022) ensures temporal independence, minimizing potential batch effects associated with single seizure events.

#### Extraction and quantification

DNA isolation was performed using the *DNeasy mericon Food Kit* (Qiagen, Valencia, CA) for small-scale samples, and the *DNeasy Plant Mini Kit* (Qiagen, Valencia, CA) for leaf and seed materials. To optimize the protocols for specific sample types and sizes, modifications were implemented. Specifically, the *DNeasy mericon Food Kit* was adapted to use $\le$20 mg of starting leaf material, and the *DNeasy Plant Mini Kit* protocol was modified to use a single seed per sample. Following isolation, the concentration of the extracted DNA was determined using the *Qubit dsDNA HS Assay Kit* (Invitrogen, Carlsbad, California, USA).

#### Sequencing

A total of 38 samples were sequenced using the Illumina NovaSeq 6000 platform, with 250 bp paired-end reads. Following sequencing, the demultiplexing of all libraries from each sequencing lane was executed using the Illumina bcl2fastq v2.20 software. Consequently, 38 FASTQ files were generated, each containing sequencing adapter-clipped reads. These files were further complemented by FastQC v0.11.9 reports, which provided comprehensive read quality metrics.

#### Alignment analysis

Sequencing yielded up to $2.27 \times 10^7$ reads, with an average of $8.3 \times 10^6$ reads per sample. In particular, all samples exhibited Phred quality scores that exceeded 30.

For alignment, the Cannabis sativa reference sequence GCF_029168945.1 (v.2.0; GenBank accession number), available from the NCBI database and representing a genome size of 770.3 Mb, was employed. This process was executed using the Bowtie2 program [23] without restrictions imposed. Following alignment, a filtering criterion was applied. Only samples demonstrating an alignment percentage greater than 50% were retained for subsequent analysis. Consequently, initially in the ".sam" format, the resulting files underwent a series of transformations. First, they were sorted and converted into the ".bam" format. Subsequently, these ".bam" sequences were transformed into a consensus sequence in ".fasta" format, which approximates the size of the reference genome. This transformation was achieved using the Samtools program [24].

#### SSR extraction

The sequences, provided in ".fasta" format, together with the reference sequence GCF_029168945.1, were processed using the Micro and Mini SATellite IdentificatioN (SATIN) program [10] to identify SSRs. In particular, this tool can delineate distinct SSR sequences based on the number of repetitions of each marker at individual loci. Furthermore, a unique feature of SATIN is its ability to preselect a subset of SSRs by performing comparative analyzes between markers and each genome. This enables the identification of the most significantly divergent SSRs in the compared groups, even in the absence of a reference genome. Following SSR identification, all pertinent features were consolidated into a tabular data set within a single comma-separated value (CSV) raw file. This file also incorporated information from the cultivation origin site for each sample, which served as labels. Consequently, this comprehensive data set functioned as input data for subsequent analytical procedures.

#### Feature Selection

**Fig. 1:**
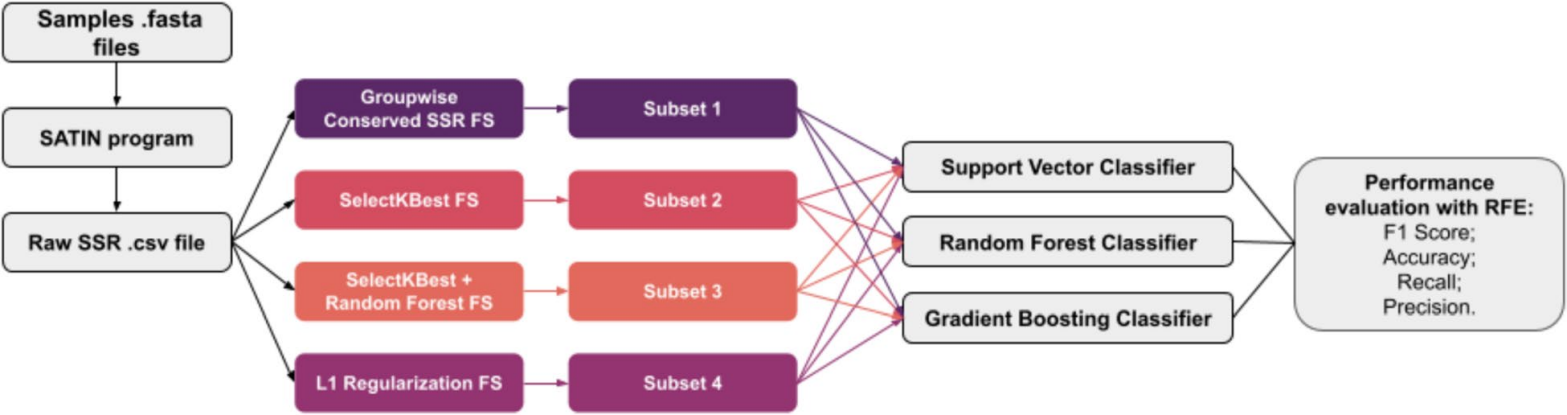
Flowchart illustrating the pipeline used for SSR extraction, FS, ML analysis and evaluation of SSR markers

Based on the generated data set, feature selection (FS) was implemented to reduce the number of irrelevant or redundant features. The FS step was integrated into our analytical pipeline (Fig. 1) and executed using four distinct strategies in Python 3.10.12, using the packages numpy v1.26.4, pandas v1.5.3, and scikit-learn v1.3.2. The objective was to determine which strategy would produce the optimal subset of SSRs from the raw CSV file for the *C. sativa* sample classification task.

#### Group-wise conserved SSR (GC-SSR) FS

The initial stage involved straightforward filtering of the total extracted SSRs. This process involved comparing samples, excluding the reference sequence, and eliminating SSR sequences containing "N" nucleotides. Only SSRs in all samples within at least one of the four origin groups (MP, FG, PG, or CO) were retained. This approach deviates from the classical feature selection (FS) methodologies found in the literature, instead representing an adaptation centered on conserving SSR markers across sample groups. Consequently, the Group-wise Conserved SSR Selection method served as a benchmark to determine the maximum number of features selected in subsequent feature selection techniques.

#### SelectKBest FS

The second method used was SelectKBest, a filter-based approach. This technique selects the top K features based on their statistical scores. Specifically, it uses the Analysis of Variance (ANOVA) F-value between labels and features for classification tasks, employing the f_classif score function. This ensures that the features with the highest discriminative power are retained, contributing to a more efficient and accurate classification model.

#### Hybrid FS

Subsequently, a two-stage hybrid FS process was implemented, combining the SelectKBest filtering method with Random Forest (RF) [5], an ensemble learning technique. This approach further incorporated hyperparameter tuning and Recursive Feature Elimination (RFE), a wrapper method. Initially, SelectKBest was applied to reduce the dimensionality of the raw SSR CSV file to a predetermined limit of K features. Following this initial reduction, RandomizedSearchCV was used to identify the optimal hyperparameters for an RF Classifier. A random search strategy, consisting of 150 iterations and four-fold cross-validation, was performed. The optimization criterion was the weighted F1 score, chosen to account for the inherent class imbalance within the dataset. Finally, RFE was performed using the optimized RF model, iteratively eliminating the least significant features to further refine the feature subset.

#### Least absolute shrinkage and selection operator FS

Finally, an embedded feature selection method was employed using a logistic regression model with L1 regularization (LASSO) [29]. A One-vs-Rest (OvR) strategy was implemented to facilitate multiclass classification, where a distinct binary classifier was trained for each class. Each classifier was designed to discriminate a single class against all others, with the overall loss function calculated as the sum of individual class losses. A *randomizedSearchCV* procedure was performed across a broad spectrum of inverse regularization values to determine the optimal regularization strength. This optimization process used Leave-One-Out Cross-Validation (LOOCV) to evaluate each parameter configuration. Subsequently, the logistic regression model was re-fitted using the identified optimal regularization strength. Following the training of the optimized model, the SelectFromModel function was applied to identify the most significant SSRs. This selection was based on the non-zero coefficients derived from the Lasso model, effectively highlighting the features with the most significant predictive power. Mathematically, the Lasso loss function, which combines cross-entropy loss with L1 regularization, is expressed as described by Eq. 1.

1 $$\begin{aligned} \text {Lasso loss} = -\frac{1}{n} \sum _{i=1}^{n} \sum _{k=1}^{K} \mathbb {1}(y_i = k) \log (p_{i,k}) + \lambda \sum _{k=1}^{K} \sum _{j=1}^{m} |\omega _{k,j}| \end{aligned}$$

Where:

- *n* is the number of samples.
- *K* is the number of classes.
- *m* is the number of features.
- $y_i$ is the true class label of the *i*-th sample.
- $p_{i,k}$ is the predicted probability that the *i*-th sample belongs to class *k*.
- $\mathbb {1}(y_i = k)$ is an indicator function that is 1 if the true class label is $y_i = k$, and 0 otherwise.
- $|\omega _{k,j}|$ are the model coefficients (weights) for class *k* and feature *j*.
- $\lambda$ is the regularization strength.
- $\sum _{k=1}^{K} \sum _{j=1}^{m} |\omega _{k,j}|$ represents the L1 regularization term, which sums the absolute values of all the model weights.

### Geographic origin classification of *C. sativa* via ML and SSR analysis

To determine the geographical origin of *C. sativa samples*, we explored and evaluated three distinct classifier algorithms for each subset of SSRs obtained in the preceding steps: Support Vector Classifier (SVC), Random Forest (RF) Classifier, and Gradient Boosting (GB) Classifier. For the SVC algorithm, a linear kernel was pre-selected. Balanced class weights were configured for both the SVC and the RF models to mitigate the impact of class imbalance.

Before model evaluation, hyperparameter tuning was performed for each SSR subset and classifier combination. This process employed Bayesian optimization with 100 iterations, using 3-fold cross-validation and the weighted F1 score as the scoring function. Subsequently, the optimized algorithms were evaluated using Leave-One-Out Cross-Validation. To identify the most informative SSRs within each subset, recursive feature elimination was used with a step size of 1. Specifically, RFE was applied to select the optimal number of features in each combination of FS method / classifier algorithm, ranging from 1 to 30 SSRs (in ascending order), at each iteration. It is important to note that the hyperparameters optimized in the previous step were held constant during the RFE process to maintain computational feasibility.

For each selected feature count, i.e., SSR added to the model generated by the algorithm, the following performance metrics were calculated 30 times to ensure robustness and statistical significance: weighted F1 score, precision, weighted recall and weighted precision. The inclusion of these distinct metrics ensures a comprehensive evaluation, allowing the models to be assessed not only by their overall balance (F1) but also by their specific ability to minimize false negatives (Recall) or false positives (Precision), depending on the specific priorities of the forensic investigation. The mean weighted F1 score values for each added SSR were plotted on a learning curve. Furthermore, and also for each combination of FS method/classifier algorithm, the average confusion matrix is also presented for the entire learning curve construction process.

### SSR-based clustering via PCoA and UPGMA

Clustering analyzes were performed using Python 3.10.12 to complement the classification modeling. These analyzes used the optimal number and combination of SSRs identified within the subset that yielded the maximum performance for each classification model. Principal Coordinate Analysis (PCoA) was performed, using the Jaccard distance metric, and implemented using the Scipy v1.11.2 and Scikit-bio v0.5.8 libraries. Subsequently, hierarchical clustering was executed. This used the Unweighted Pair Group Method with Arithmetic Mean (UPGMA) algorithm, also based on the Jaccard distance, and was performed using the Scipy v1.11.2 library.

## Experiments and results

The alignment of the 38 genomes to the reference genome yielded an average alignment rate of 90.465 ± 1.268%. The aligned sequences were subsequently processed, leading to the identification of 467,527 SSRs in a raw SSR dataset. This dataset served as input for the FS methods, generating their respective subsets, which were later used in classification tasks across different supervised learning models (Fig. 1). The implemented pipeline resulted in 12 distinct combinations of FS strategies coupled with ML classifiers. A more detailed description of the SSRs present in each subset generated from the raw SSR file can be found in Table S2 of the Supplemental Material.

Regarding the GC-SSR FS, following filtering steps - including intersample comparisons and removing SSRs containing nucleotide N -, 281 SSRs within coding sequences were selected. Of these, 97.49% were classified as perfect sequences, 2.49% as non-perfect sequences, and 7 as complex sequences. Among perfect sequences, dinucleotide repeats were the most prevalent, accounting for more than 48%, followed by mononucleotide repeats in 20% and trinucleotide repeats in 16.7%. Regarding the di-nucleotide composition, AG repeats were observed in the highest proportion, followed by TC, AT, and CT repeats. Retaining only those SSRs conserved across all samples within at least one origin group resulted in the remaining 30 SSRs (Subset 1). Except for the loci LOC115707685 and LOC115707221, each possessing two markers, all other SSRs were located at distinct loci. Of these 30 SSRs, 18 were identified in the genomes of the CO origin group, with 9 exclusive to this region: (GAT)4, (T)12, (TC)21, (A)10, (T)11, (GAAT)3, (GAA)4, (TCTTCG)3, (A)16. Similarly, 16 SSRs were found in the MP origin group, with 6 exclusive: (ATC)5, (CT)5, (TAA)4, (A)11, (CACCAA)3, (CGAAGA)3. In the FG origin group, 8 SSRs were identified, with 4 exclusive: (GA)5, (AGC)6, (TCA)5, (GAA)4. In particular, SSR (AAG) 5 was present in all regions. In contrast, no conserved SSR was identified in all genomes within the PG origin group.

Given that the quantity of SSRs selected by subsequent feature selection (FS) strategies is arbitrary, the Group-wise Conserved SSR selection (which naturally yielded 30 markers) was used as a benchmark to establish an upper threshold of 30 SSRs for the other subsets. It is important to note that this threshold served only as an initial ceiling for comparative purposes; the final number of markers included in the predictive models was dynamically determined through Recursive Feature Elimination, which optimized the feature set based on model performance metrics.

For subsets 2 and 4, 30 SSRs exhibiting the highest statistical relevance were selected. This selection was achieved through the application of the SelectKBest and Lasso methods, prioritizing the ANOVA F-values and the nonzero coefficient values, respectively. With the exceptions of (A)21 and (T)21 in LOC115705433, and (CA)5 and (CG)5 in LOC115707672 for subset 2, as well as (CAC)4 and (TGTT)3 in LOC115705474 for subset 4, all other extracted SSR markers were located at different loci within each subset. Lastly, for subset 3, the reduction in dimensionality using the SelectKBest technique yielded the 5,000 most relevant SSRs from the raw file. Subsequently, these SSRs were then used as input for a Random Forest (RF) Classifier, from which 30 SSRs were selected based on feature importance derived from the mean decrease in impurity of decision trees, utilizing the Recursive Feature Elimination (RFE) technique. Again, with the exception of (GA)5 and (TC)5 in LOC115705181, all other SSRs were present at different loci within this subset. Comparative analysis revealed that subsets 2, 3, and 4 shared the following selected SSRs: (ATT)4 at the LOC115702144, (T)10 at the LOC133031214 and (TCA)4 at the LOC115704795 locus. Similarly, subsets 1, 2, and 4 shared markers (GAAT)3 at locus LOC115706559, (TCA)5 at locus LOC133034467, and (CACCAA)3 at locus LOC115707482. Furthermore, the following SSRs were present in at least two different subsets: LOC115704143 (GGAAA)3, LOC115704734 (TCC)4, LOC115705161 (TGG)5, LOC115705474 (TGTT)3,

LOC115706366 (T)11, LOC115707221 (GAT)4,

LOC115707563 (TCT)4, LOC115707882 (AT)5,

LOC115708333 (A)16 and LOC133035775 (GGAG)3.

The choice criterion for defining the best combination of the FS strategy and the classification algorithm was based on a trade-off between the maximum score achieved during the learning curve and the number of SSRs incorporated. This selection strategy effectively assigns equal weight to model accuracy and model parsimony, aligning with multi-objective optimization principles. By prioritizing the solution that offers the highest performance with the fewest features, we aim to minimize the risk of overfitting and facilitate the practical implementation of smaller, more cost-effective multiplex panels in forensic laboratories. Of all distinct combinations, optimal performance with the fewest markers was achieved using subset 1 within a Gradient Boosting model (Table 3 and Fig. S4, Supplementary material). Using a combination of 7 SSRs from the original Subset 1, the model successfully distinguished all samples based on their origins (Fig. S4B), demonstrating a mean F1 score, precision (Acc), precision (Prec), and recall (Rec) of 1.0. These SSRs include LOC133034467 (TCA)5, LOC115706559 (GAAT)3, LOC115705224 (TAA)4, LOC115706366 (T)11, LOC115707030 (GA)5, LOC115707832 (AAG)5, and

LOC115707482 (CACCAA)3. Conversely, the other Gradient Boosting models generated from Subsets 2, 3, and 4 exhibited the lowest scores among all tested combinations, ranked in descending order: Subset 3 (F1: 0.92, Acc: 0.92, Prec: 0.93, Rec: 0.92) using 23 SSRs, Subset 4 (F1: 0.83, Acc: 0.84, Prec: 0.84, Rec: 0.84) using 3 SSRs, and Subset 2 (F1: 0.81, Acc: 0.81, Prec: 0.82, Rec: 0.81) using 8 SSRs (Figs. S5, S6 and S7, Supplementary material).

**Fig. 2:**
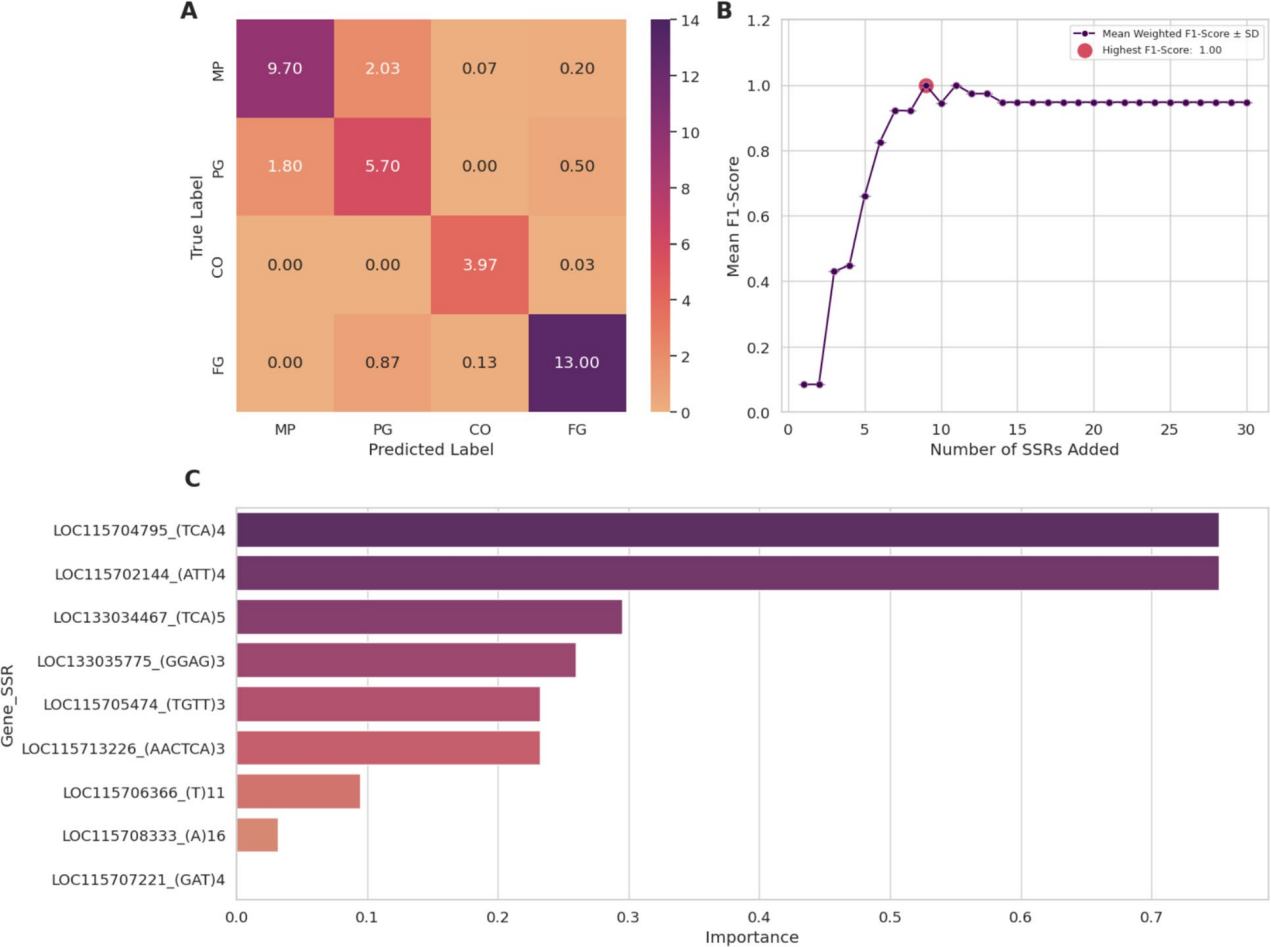
Evaluation of the Linear SVC Model using Subset 2 SSRs Selected by SelectKBest Feature Selection. (A) The average confusion matrix across all replicates is presented, demonstrating the classification performance for the four sample origins: MP, PG, CO, and FG. (B) The weighted F1-score learning curve, including standard deviations, is depicted as a function of the number of added SSR markers. The red marker indicates the maximum observed F1-score. (C) Feature importances are shown for the 9 SSRs utilized in the Linear SVC model that achieved the highest score

The SVC generated models demonstrated superior overall performance across all four subsets, requiring only a few additional Simple Sequence Repeats (SSRs) for classification (Table 1). In particular, using only 9 SSRs from Subset 2, we achieved a maximum F1 score of 1.0 (Fig. 2 - B). These SSRs, in order of importance for the model (Fig. 2 - C), were as follows: LOC115704795 (TCA)4, LOC115702144 (ATT)4, LOC133034467 (TCA)5, LOC133035775 (GGAG)3, LOC115705474 (TGTT)3,

LOC115713226 (AACTCA)3, LOC115706366 (T)11,

LOC115708333 (A)16, and LOC115707221 (GAT)4. Subsets 1 and 4 exhibited identical performance with 10 and 11 SSRs, respectively. Subset 3 showed slightly lower classification performance using 12 SSRs (F1: 0.97; Accuracy: 0.97, Precision: 0.98, Recall: 0.97).

RF classifiers also showed high predictive ability (Table 2), although no subset reached the maximum possible score. The scores obtained for each subset in this classification model were in decreasing order of performance: Subset 1 (F1: 0.97, Acc: 0.97, Prec: 0.97, Rec: 0.97) using 14 SSRs, Subset 3 (F1: 0.95, Acc: 0.95, Prec: 0.95, Rec: 0.95) using 22 SSRs, Subset 2 (F1: 0.92, Acc: 0.92, Prec: 0.92, Rec: 0.92) using 14 SSRs and Subset 4 (F1: 0.85, Acc: 0.87, Prec: 0.85, Rec: 0.87) using 14 SSRs. RF classifiers learning curves, features importances and confusion matrices can also be found in supplementary material (Figs. S8-11).

**Table 1:** Number of SSRs incorporated in the model, Mean Weighted F1-score (F1), Mean Accuracy (Acc), Mean Weighted Precision (Prec), and Mean Weighted Recall (Rec) metrics with their respective standard deviations (SD) calculated from 30 replicates for different subsets using Linear SVC. Subset with the best performance using the respective algorithm is marked with an asterisk (*)

	Linear SVC				
	SSRs incorp.	Mean F1±SD	Mean Acc±SD	Mean Prec±SD	Mean Rec±SD
Subset 1	10	1.0±0.0	1.0±0.0	1.0±0.0	1.0±0.0
Subset 2$^*$	9	1.0±0.0	1.0±0.0	1.0±0.0	1.0±0.0
Subset 3	12	0.97±1.11e-16	0.97±2.22e-16	0.98±2.22e-16	0.97±2.22e-16
Subset 4	11	1.0±0.0	1.0±0.0	1.0±0.0	1.0±0.0

**Table 2:** Number of SSRs incorporated in the model, Mean Weighted F1-score (F1), Mean Accuracy (Acc), Mean Weighted Precision (Prec), and Mean Weighted Recall (Rec) metrics with their respective standard deviations (SD), calculated from 30 replicates for different subsets using the Random Forest (RF) classifier. Subset with the best performance using the respective algorithm is marked with an asterisk (*)

	Random Forest				
	SSRs incorp.	Mean F1±SD	Mean Acc±SD	Mean Prec±SD	Mean Rec±SD
Subset 1$^*$	14	0.97±0.02	0.97±0.02	0.97±0.02	0.97±0.02
Subset 2	14	0.92±0.02	0.92±0.02	0.92±0.02	0.92±0.02
Subset 3	22	0.94±0.03	0.94±0.03	0.94±0.03	0.94±0.03
Subset 4	14	0.85±0.03	0.87±0.02	0.85±0.04	0.87±0.02

**Table 3:** Number of SSRs incorporated in the model, Mean Weighted F1-score (F1), Mean Accuracy (Acc), Mean Weighted Precision (Prec), and Mean Weighted Recall (Rec) metrics with their respective standard deviations (SD) calculated from 30 replicates for different subsets using the Gradient Boosting (GB) classifier. Subset with the best performance using the respective algorithm is marked with an asterisk (*)

	Gradient Boosting				
	SSRs incorp.	Mean F1±SD	Mean Acc±SD	Mean Prec±SD	Mean Rec±SD
Subset 1$^*$	7	1.0±0.0	1.0±0.0	1.0±0.0	1.0±0.0
Subset 2	8	0.81±0.03	0.81±0.03	0.82±0.03	0.81±0.03
Subset 3	23	0.92±0.02	0.92±0.02	0.93±0.02	0.92±0.02
Subset 4	3	0.83±0.06	0.84±0.06	0.83±0.07	0.83±0.06

## Discussion

### Feature selection

Advances in molecular biology technologies have led to an exponential increase in biological data available in recent decades, leading to the large p, small n issue, a very common scenario in life science studies where the number of observations or samples (n) is hundreds or thousands of times smaller than the number of markers or characteristics extracted (p). Within the field of ML, this can be translated into what we call the curse of dimensionality, that is, as dimensionality (p) increases, the number of data points (n) required for good performance of any ML algorithm increases exponentially [3]. In addition to the problem of high-dimensional data, another obstacle that can affect the performance of ML models is sparsity, which refers to the high number of noninformative features that have a small or insignificant effect on the characteristic of interest, leading to an unstable predictive model [2]. Therefore, the use of FS algorithms as a means of dimensionality reduction is a crucial step in bioinformatic pipelines applied to different omics datasets. In the present work, we explore four different SSRs filtering strategies: GC-SSR Selection, a personalized adaptation that served as a reference technique to determine the maximum number of selected markers, in addition to filter (SelectKBest), hybrid(RF associated with RFE) and embedded (LASSO) methods for FS, obtaining four partially distinct subsets of selected SSRs.

Surprisingly, subset 1 (GC-SSR FS) achieved the best performance in predicting the origin of the sample in two of three models tested compared to the other subsets (Tables 1, 2 and 3). Although it is a form of adapted selection based on biological intuition without the application of formal statistical analysis or the complexity of a model-based FS method, GC-SSR presented the most informative set of SSRs to separate samples regarding their origin of culture, likely indicating that these markers represent genomic regions that may be functionally important and evolutionarily conserved. While we hypothesize that this conservation implies functional constraints or selective pressure, we did not explicitly quantify evolutionary rates (e.g., dN/dS ratios) in this study. This decision was based on the primary focus on forensic discrimination and the current challenges regarding the precise functional annotation of these loci in diverse illicit strains. Since these SSRs were filtered to be present in all samples from at least one of the origin groups (MP, FG, PG or CO), they could reflect genetic stability or selection for traits specific to certain populations. Such features may be more biologically meaningful than other subsets. This subset might inherently carry less noise, which is beneficial for model performance, especially for classifiers like SVC with a linear kernel or RF, which might be sensitive to noisy or irrelevant features. On the other hand, methods like SelectKBest purely on statistical metrics and may retain features that, while statistically significant, are not as biologically informative.

**Fig. 3:**
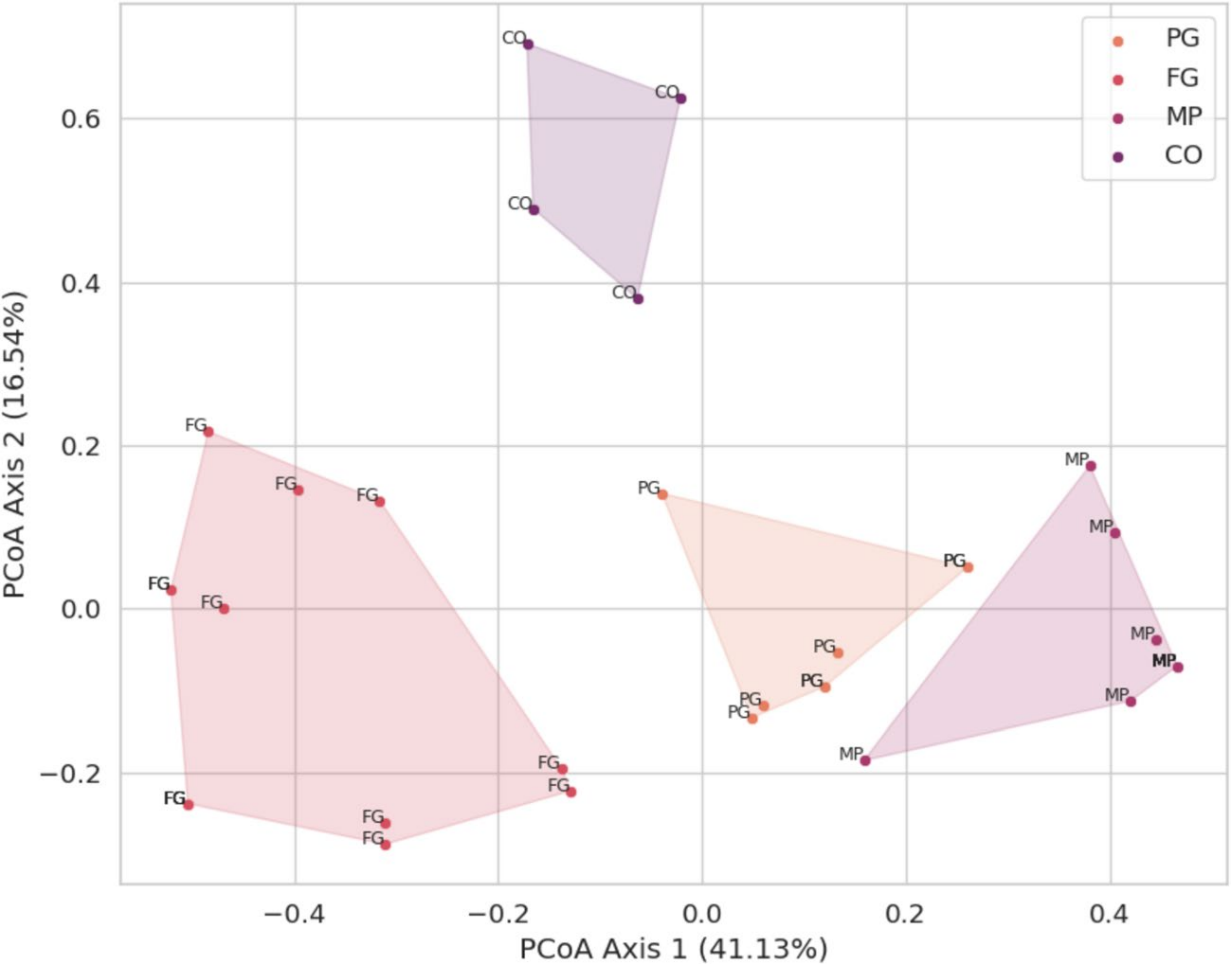
**Principal Coordinate Analysis (PCoA) plot based on Jaccard distance calculated from the 9 SSR markers used in SVC best model.** The plot shows the ordination of samples across two dimensions (PC1 and PC2), which together explain 41.13% and 16.54% of the variation, respectively

Regarding subsets 3 (Hybrid FS) and 4 (LASSO FS), there are some combinations of factors that may justify their lower performance in certain models. Despite being a popular solution for dimensionality reduction, LASSO can be overly aggressive in feature elimination and tends to select only one feature from a group of highly correlated features. Although this can be beneficial in highly correlated or high-dimensional datasets, it can result in the loss of important features that are useful for classification [38]. As for the hybrid FS method, which combines filter (SelectKBest), ensemble (RF), and wrapper (RFE) approaches, the greater degree of complexity introduced in the attribute selection task may not always result in better performance, particularly in small and imbalanced datasets. Also, specifically in the RFE-associated RF application stage, if the hyperparameters were not optimized perfectly, the performance of the hybrid FS method might not reach its full potential since this model is more sensitive to hyperparameter tuning.

Furthermore, it is important to clarify that our approach treated each SSR motif as an independent feature during the selection process, even when multiple motifs originated from the same locus (e.g., LOC115705433 in Subset 2). We did not manually merge or filter these features; instead, we relied on the FS algorithms to determine their individual discriminative power. The selection of multiple motifs from a single locus by methods like SelectKBest suggests that these specific repeats may provide complementary information. It is possible that these markers exhibit linkage disequilibrium or distinct mutational patterns that, when combined, enhance the statistical separation of the sample groups.

### Classification task and origin prediction

As for the task of predicting the origin and classifying samples of *C. Sativa*, the highlight goes to the linear kernel SVC, which presented excellent performance regardless of the subset of SSRs used (Table 1). These results indicate that, regardless of the FS method used, SSRs are extremely discriminative markers and provide practically perfect linear separability, using a simpler classification model, with low computational cost and easily interpretable when compared to RF and GB. The combination of subset 2 associated with SVC proved to be the most efficient, performing the task with only 9 SSRs.

The 9 SSRs are located at different protein-coding loci on chromosome 1. LOC115704795 (TCA)4, present in samples from MP, encodes for *CsNAC01*, which is part of the NAC transcription factor gene family, related to direct and indirect regulation of response to abiotic stresses, such as low temperatures, excessive heat, drought, etc. [25, 45]. LOC115702144 (ATT)4, also present in MP samples, and LOC115707221 (GAT)4, present in CO samples, encode the putative ubiquitin protein ligase E3 LIN-1 and ATL42, respectively, which are members of the E3 Ubiquitin Ligases family, a conserved group of enzymes among eukaryotes responsible for a range of regulatory functions that aid in covalent binding of ubiquitin to target proteins [21]. LOC115705474 (TGTT)3, present in MP and PG samples, encodes the F-box protein CPR1, which is responsible for negatively regulating plant immunity by promoting the degradation of resistance proteins (NLR), such as SNC1 and RPS2, through the ubiquitin proteasome system, essential to avoid excessive activation of immune responses, which can lead to impaired plant growth and autoimmunity [7, 17]. LOC115708333 (A)16, present in CO samples, encodes a probable intermediate-associated protein of complex I 30 (CIA30) or NADH dehydrogenase ubiquinone 1 alpha subcomplex assembly factor 1 (NDUFAF1), an important assembly factor of mitochondrial complex I, the largest protein complex operating in oxidative phosphorylation [37, 44]. LOC133034467 (TCA)5 and LOC133035775 (GGAG)3, present in samples from FG and some samples from the PG region, as well as LOC115713226 (AACTCA)3, present in MP and PG samples, and LOC115706366 (T)11 present in CO samples, are located in *C. sativa* uncharacterized genome regions. A plausible hypothesis is that these SSRs are a consequence of adaptive mutations important for the adaptation of *C. sativa* to different cultivation environments, such as the regulation of the response to environmental stresses, modulation of plant immunity, and metabolic and energetic adaptability of the plant, but additional studies would be necessary to prove such assumptions. In addition, because of the presence of several loci that have not yet been characterized, it is possible that there is some hidden correlation that has not yet been studied.

**Fig. 4:**
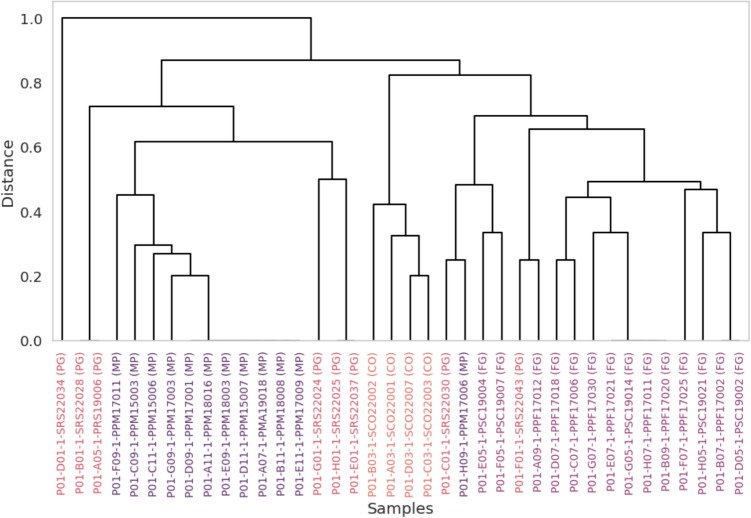
Hierarchical clustering dendrogram based on UPGMA method and Jaccard distance. The dendrogram illustrates the genetic relationships among samples using the 9 SSR markers used in SVC best model. The samples are color-coded according to their origin, with MP in purple, PG in red, CO in orange-red, and FG in darker purple

Combinations involving GB classification models obtained, on average, a lower performance than the others (Table 3). Subset 2 and subset 4 associated with GB obtained metrics just above 0.80 at their peak performance, despite using only 8 and 3 SSRs to classify the samples, respectively. Subset 3 associated with GB obtained a good peak performance above 0.90 for all metrics, but required 23 SSRs to achieve this score, a higher amount than the forensic microsatellite panels already validated and used for the identification and individualization of samples of Brazilian *C. sativa* in previous work [14, 30]. Subset 1 associated with the GB model, on the other hand, obtained a perfect score and correctly predicted the origin of all samples using only 7 SSRs (Fig. 4). Again, the 7 SSRs used for classification are also located at different loci of chromosome 1. LOC133034467 (TCA)5, present in MP samples, and LOC115706559 (GAAT)3/LOC115706366 (T)11, present in CO samples, are in uncharacterized coding regions. LOC115705224 (TAA)4, present in MP samples, encodes the late elongated hypocotyl protein (LHY), which regulates photoperiodic flowering through the circadian clock in *Arabidopsis thaliana* [31, 47]. LOC115707030 (GA)5, present in samples originating from the FG and MP groups, encodes Myosin XVII, which is part of a large family of motor proteins involved in various processes in eukaryotic cells [27, 39]. LOC115707832 (AAG)5 encodes the Rho proteins of plants (ROPs) guanine nucleotide exchange factor 12, involved in sending signals to downstream cellular targets, and is curiously present in practically all samples, with the exception of some from PG, which, added to its relatively low relevance within the model (Fig. S4C, Supplementary material), suggests that it was used to classify a specific sample within the group [4, 13, 48]. The LOC115707482 (CACCAA)3, present in MP and CO samples, encodes the early flowering protein MYB, a transcription factor responsible for regulating the flowering process in response to changes in light and temperature in *Arabidopsis thaliana* [18, 46]. Although the GB model also achieved a perfect score and used the lowest number of SSRs among all combinations tested, its inferior performance for the other subsets suggests a greater dependence on specific characteristics to achieve high performance. This indicates greater instability and possibly lower generalizability compared to SVC. Furthermore, GB models require higher computational power and greater hyperparameter tuning.

Interestingly, samples from the PG region did not present any SSRs that were present in all samples from the group, but rather were distributed among a few representatives. Because of this, the amount of SSRs required to predict the place of origin of samples from this group in all classifiers was considerably higher when compared to those from MP, FG, and CO. This characteristic indicates a greater intragroup genetic diversity, possibly caused by factors such as variation in cultivation practices, different subpopulations within the same location, or the influence of varied environmental conditions, and may lead to a lower consistency between SSR markers among samples from the region, thus requiring more markers to represent the greater genetic heterogeneity present. Consequently, in the GC-SSR analysis (Subset 1), the PG samples were classified not by their own unique conserved markers, but by their distinct profile across the markers defined by the MP, CO, and FG groups. The classifiers effectively leveraged the specific absence or variable frequency of these inter-group conserved markers to distinguish the PG samples, demonstrating that a panel derived from specific groups can still be useful for identifying samples from diverse, highly heterozygous origins.

### Clustering analysis

PCoA and hierarchical clustering were performed for each model using the combination and the number of SSRs from the subset with which they reached their peak performance, according to Tables 1, 2 and 3. Again, the 9 SSRs from subset 2 used by the SVC model were more prominent and could visually distinguish the four seizure groups according to their biogeographic origin (Figs. 3 and 4). PCoA and hierarchical clustering analysis related to the 7 and 14 most informative SSRs from Subset 1 applied to the models generated by GB and RF, respectively, can be found in the supplementary material (Figs. S12, S13, S14 and S15).

In PCoA based on SSRs used by the best model obtained with the SVC classifier (Fig. 3), the principal coordinates Coord.1 and Coord.2 explained approximately 41.13% and 16.54%, respectively, of the genetic variance explained, based on the Jaccard distance matrix. Interestingly, samples belonging to the foreign group (FG), coming from seeds of different commercial strains seized from postal services, formed a group spread across the X and Y axes. BFP reports suggest that the seeds came from Europe, although the exact location of the origin could not be determined. This dispersion in the PCoA likely reflects the high genetic diversity and admixture characteristic of commercial hybrid strains. Unlike the geographically distinct groups, the FG samples lack a single, defined biological origin, representing instead a mixture of diverse genotypes entering the country. We acknowledge that this heterogeneity limits the interpretation of clustering patterns for this specific group; however, it accurately represents the forensic reality of the ’foreign’ class in the Brazilian illegal market, which is composed of a complex variety of commercial strains rather than a single lineage.

Meanwhile, hierarchical cluster analysis (Fig. 4) based on the Jaccard distance revealed that while the FG, MP and CO groups formed distinct, well-defined clusters, samples from the Paraguay group (PG) were scattered among these clusters. This pattern likely indicates higher genetic diversity or a more heterogeneous genetic structure within the PG group and reinforces the results found during the evaluation of the classification models, which show the absence of distinctive and informative SSR markers in the samples. This heterogeneity is consistent with the origin of these samples: they were derived from large-scale ’pressed brick’ seizures intercepted along major entry routes (e.g., Mato Grosso do Sul and Paraná). Unlike single-site cultivations, these shipments likely represent an aggregation of harvested material from multiple distinct farms or regions within Paraguay, resulting in a highly admixed genetic profile that lacks the fixed markers observed in more localized groups.

## Conclusion

Our study successfully identified informative SSR markers capable of distinguishing *C. sativa* samples from defined geographic regions, as well as samples of uncertain provenance (FG), which are potentially related to trafficking routes supplying the illegal Brazilian market. Using simple filtering and feature selection techniques, associated with classification models widely used in the literature, with emphasis on linear kernel SVC, we obtained different panels of SSR markers with high predictive value and discriminative power. However, it is important to highlight the need for larger and balanced sample databases to evaluate the true discriminatory power of these markers and possibly discover and incorporate more informative markers for Brazilian *C. sativa* samples, also reducing the risk of overfitting. Specifically, regarding the limitation observed in the commercial strains (FG), future research must prioritize the use of plant samples with well-documented and certified origins. Establishing a ’ground truth’ with certified samples will be crucial to obtaining more robust and meaningful results and to refining the identification of complex hybrid strains in practical investigative settings.

Although the results obtained are promising, highlighting the usefulness of these markers in the context of supervised ML, additional studies are necessary in new sample groups for experimental validation on the bench to validate their application in practical investigative settings. This would also allow the investigation of several forensic quality parameters commonly evaluated in these types of markers, such as the mean number of alleles, the effective number of alleles, the probability of random match, the power of exclusion and the power of discrimination, and population differentiation metrics such as the Fixation Index ($F_{ST}$), ideal for forensic and law enforcement purposes.

## Supplementary Information

Below is the link to the electronic supplementary material.

Supplementary file 1 (pdf 1728 KB)
