# Supplementary material for: Identification of biogeographically informative microssatelite markers for Brazilian Cannabis sativa samples: a machine learning approach for forensic origin prediction
Source: Int J Legal Med. 2026 Feb 6;140(3):1313–25. doi: 10.1007/s00414-025-03716-7 (PMC13160992; doi:10.1007/s00414-025-03716-7)
Supplement: Supplementary file 1 — (pdf 1728 KB) [file 414_2025_3716_MOESM1_ESM.pdf]

Supplementary Material

Group name	N	Aphreension	Year	Material	Origin
Paraguay	8	Postal sorting	2019, 2022	Seed	Paraguay
Colombia	4	Postal sorting	2022	Seed	Colombia
Marijuana Polygon	12	Marijuana Polygon	2015, 2017, 2018, 2019	Leaves	Brazilian Northeastern
Foreign Group	14	Postal sorting	2017, 2019	Leaves	Unspecified

Table S1: Sample characterization

Subset 1 (GC-SSR FS)	Subset 2 (SelectKBest FS)	Subset 3 (Hybrid FS)	Subset 4 (LASSO FS)
LOC133033767_(T)12	LOC115703695_(A)10*	LOC133033509_(ACCCAA)3	LOC133034467_(TCA)5
LOC115707685_(AGTTGT)3	LOC115704143_(GGAAA)3	LOC115705090_(CGAAGA)3	LOC115705161_(TGG)5
LOC115708111_(AAT)4	LOC115704438_(T)13	LOC115702149_(AG)5	LOC115702088_(A)10
LOC115706980_(AGCT)3	LOC115704528_(CA)5	LOC115702144_(ATT)4	LOC115703562_(GTT)4
LOC115706244_(A)10	LOC115704734_(TCC)4	LOC115708011_(CTAG)3	LOC115707737_(TGG)6
LOC115707803_(GA)5	LOC115704795_(TCA)4	LOC115707953_(T)13	LOC115704143_(GGAAA)3
LOC115716967_(AGG)5	LOC115705161_(TGG)5	LOC115705673_(AGAA)3	LOC115713226_(AACTCA)3
LOC115707221_(TC)21	LOC115705433_(A)21	LOC115703632_(TTCC)3	LOC115702144_(ATT)4
LOC115707221_(GAT)4	LOC115705433_(T)21	LOC115705108_(GA)5	LOC115705090_(CGAAGA)3
LOC115705075_(GAA)4	LOC115705474_(TGTT)3	LOC133031214_(T)10	LOC115705620_(AGC)6
LOC115704543_(GAA)4	LOC115705649_(T)12	LOC115704829_(A)10	LOC115706569_(TA)6
LOC115708223_(TCTTCG)3	LOC115705749_(T)10	LOC115706082_(AG)9	LOC133035775_(GGAG)3
LOC133034467_(TCA)4	LOC115706366_(T)11	LOC115704795_(TCA)4	LOC115707802_(CT)6
LOC115706366_(T)11	LOC115706559_(GAAT)3	LOC115706244_(A)10	LOC115708345_(TCA)7
LOC115705224_(TAA)4	LOC115706956_(AG)16	LOC115706065_(A)11	LOC115704734_(TCC)4
LOC115707685_(ACAACT)3	LOC115707204_(GAA)4*	LOC115705181_(GA)5	LOC115707474_(CAC)4
LOC115706559_(GAAT)3	LOC115707221_(GAT)4	LOC115707122_(A)10	LOC115707987_(TCT)4
LOC115707899_(CAC)4	LOC115707482_(CACCAA)3	LOC115705181_(TC)5	LOC115705529_(GATG)3
LOC115707482_(CACCAA)3	LOC115707563_(TCT)4	LOC115706572_(T)10	LOC115706559_(GAAT)3
LOC133034467_(TCA)5	LOC115707632_(TG)6	LOC115705166_(A)14	LOC115705224_(TAA)4
LOC133031660_(CT)5	LOC115707672_(CA)5	LOC115702149_(CT)5	LOC115707563_(TCT)4
LOC115707030_(GA)5	LOC115707672_(TG)5	LOC115705105_(TTG)7	LOC115705474_(TGTT)3
LOC115705620_(AGC)6	LOC115707743_(T)16	LOC133033866_(T)12	LOC133031214_(T)10
LOC115707832_(AAG)5	LOC115707882_(AT)5	LOC133036599_(T)18	LOC115705455_(AAC)4
LOC115705045_(T)10	LOC115708333_(A)16	LOC115708050_(T)12	LOC115707482_(CACCAA)3
LOC115705315_(T)10	LOC115713226_(AACTCA)3	LOC115707977_(A)10	LOC115706470_(ATC)5
LOC115706470_(ATC)5	LOC133031214_(T)10	LOC133033580_(A)24	LOC133032923_(ACG)4
LOC115705090_(CGAAGA)3	LOC133034467_(TCA)5	LOC115708288_(AG)5	LOC115704308_(AAC)5
LOC115708333_(A)16	LOC133035775_(GGAG)3	LOC115703771_(TC)6	LOC115707030_(GA)5

Table S2: Selected 30 SSRs panels (Subsets 1 to 4) for each FS method used. Markers represented as follow: Gene name_(SSR motif) number of repetitions.

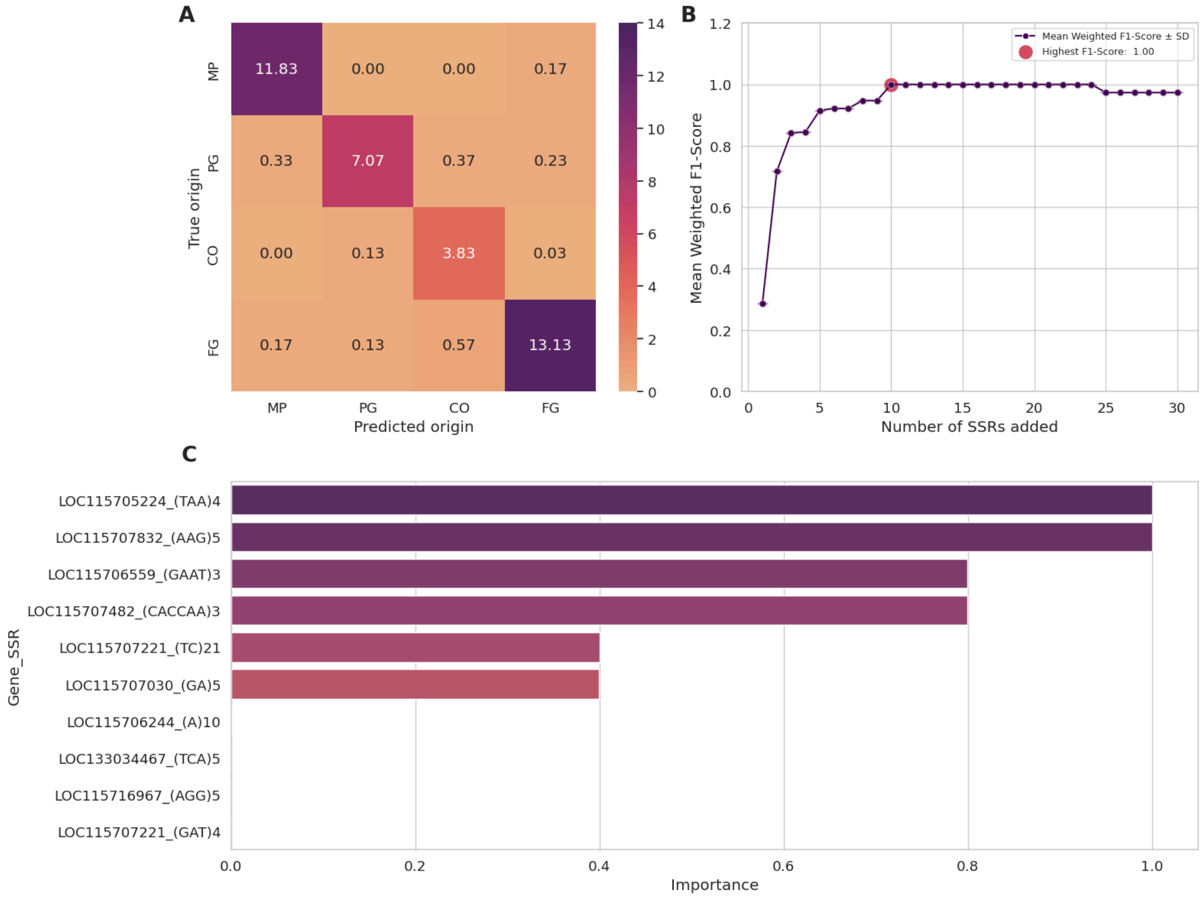

Figure S1. Evaluation of the SVC Model using Subset 1 selected SSRs (GC-SSR FS). (A) Shows the average confusion matrix across all replicas, illustrating the classification performance across four sample origins: MP, PG, CO, and FG. (B) F1-score learning curve as a function of the number of SSR markers added. The curve depicts the mean weighted F1-scores (with standard deviations) across different feature sets. The red point marks the highest observed F1-score.(C) Feature importances of the 10 SSRs used for classification task in the SVC model with highest score

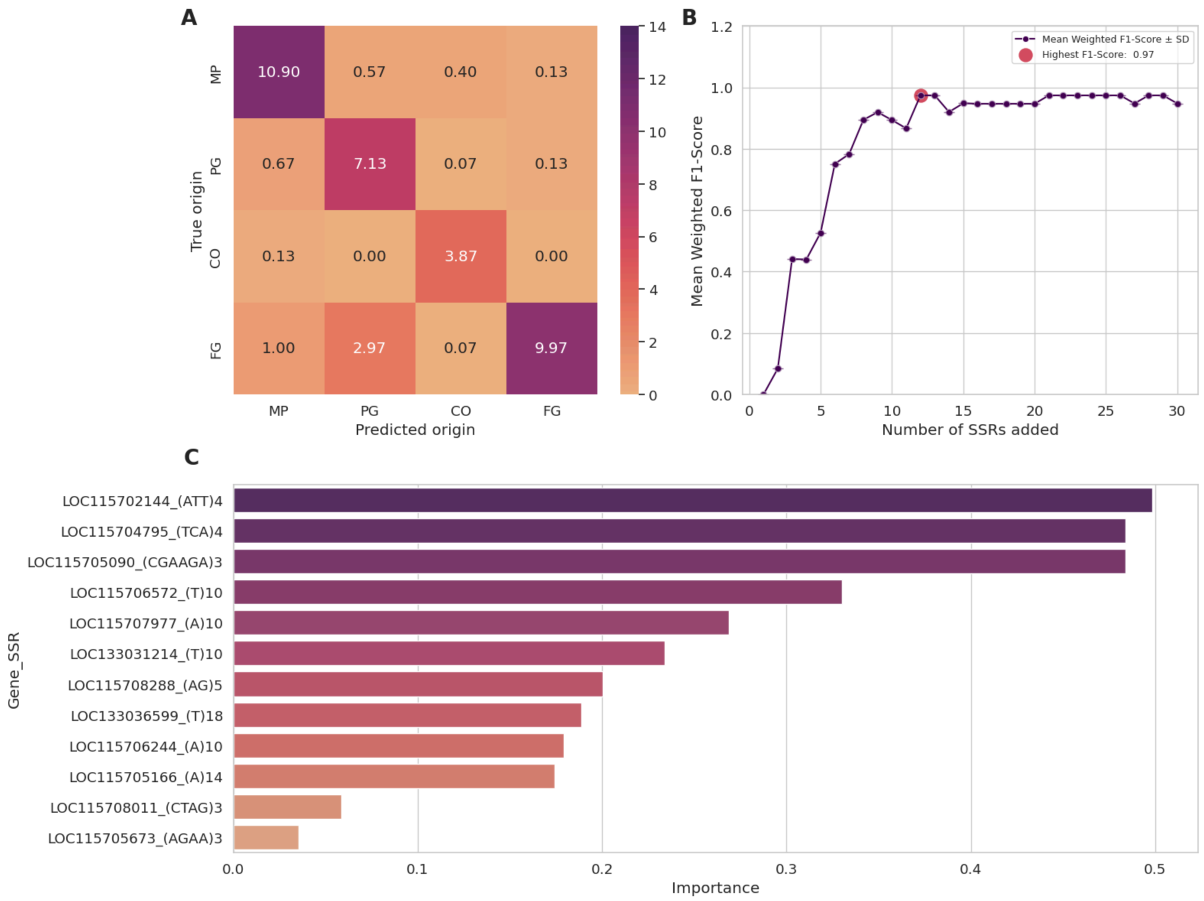

Figure S2. Evaluation of the SVC Model using Subset 3 selected SSRs (Hybrid FS). (A) Shows the average confusion matrix across all replicas, illustrating the classification performance across four sample origins: MP, PG, CO, and FG. (B) F1-score learning curve as a function of the number of SSR markers added. The curve depicts the mean weighted F1-scores (with standard deviations) across different feature sets. The red point marks the highest observed F1-score.(C) Feature importances of the 12 SSRs used for classification task in the SVC model with highest score

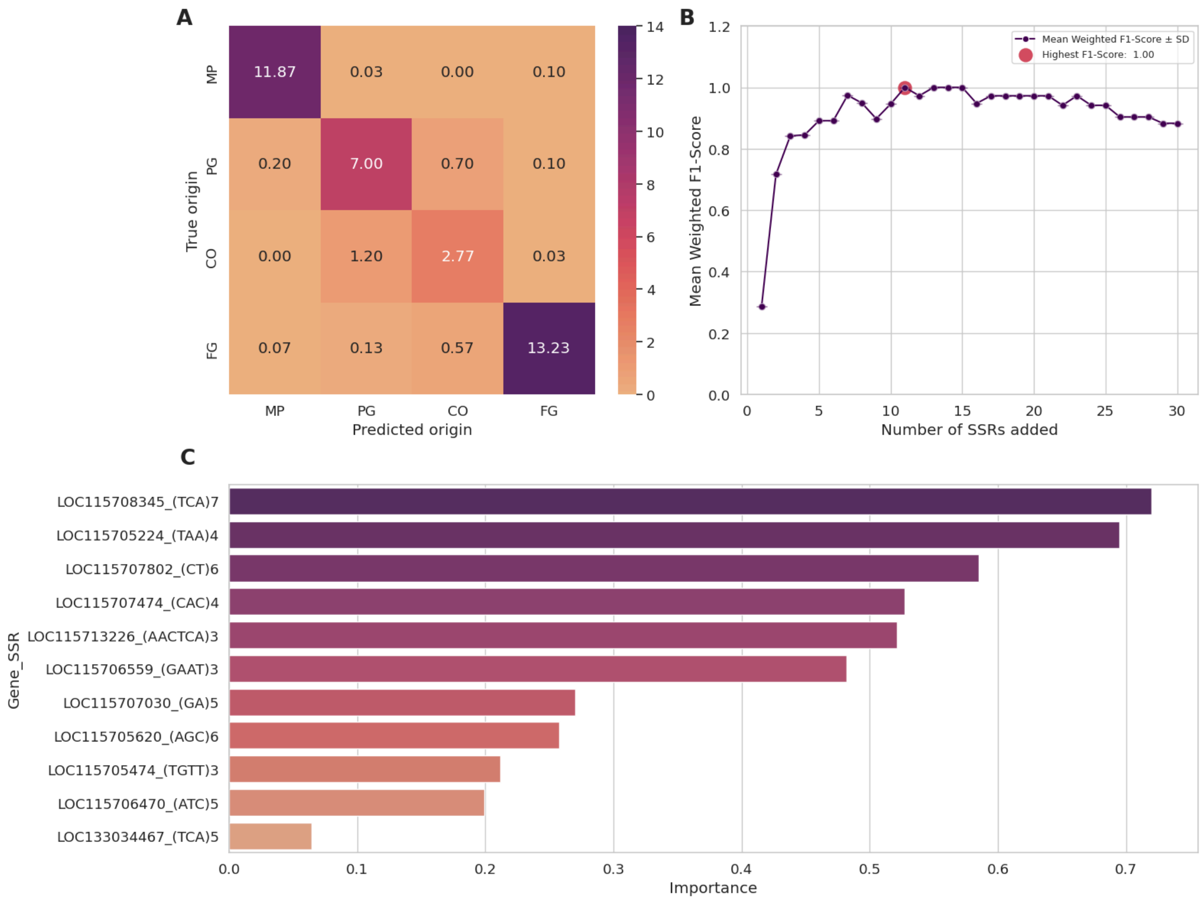

Figure S3. Evaluation of the SVC Model using Subset 4 selected SSRs (LASSO FS). (A) Shows the average confusion matrix across all replicas, illustrating the classification performance across four sample origins: MP, PG, CO, and FG. (B) F1-score learning curve as a function of the number of SSR markers added. The curve depicts the mean weighted F1-scores (with standard deviations) across different feature sets. The red point marks the highest observed F1-score.(C) Feature importances of the 11 SSRs used for classification task in the SVC model with highest score

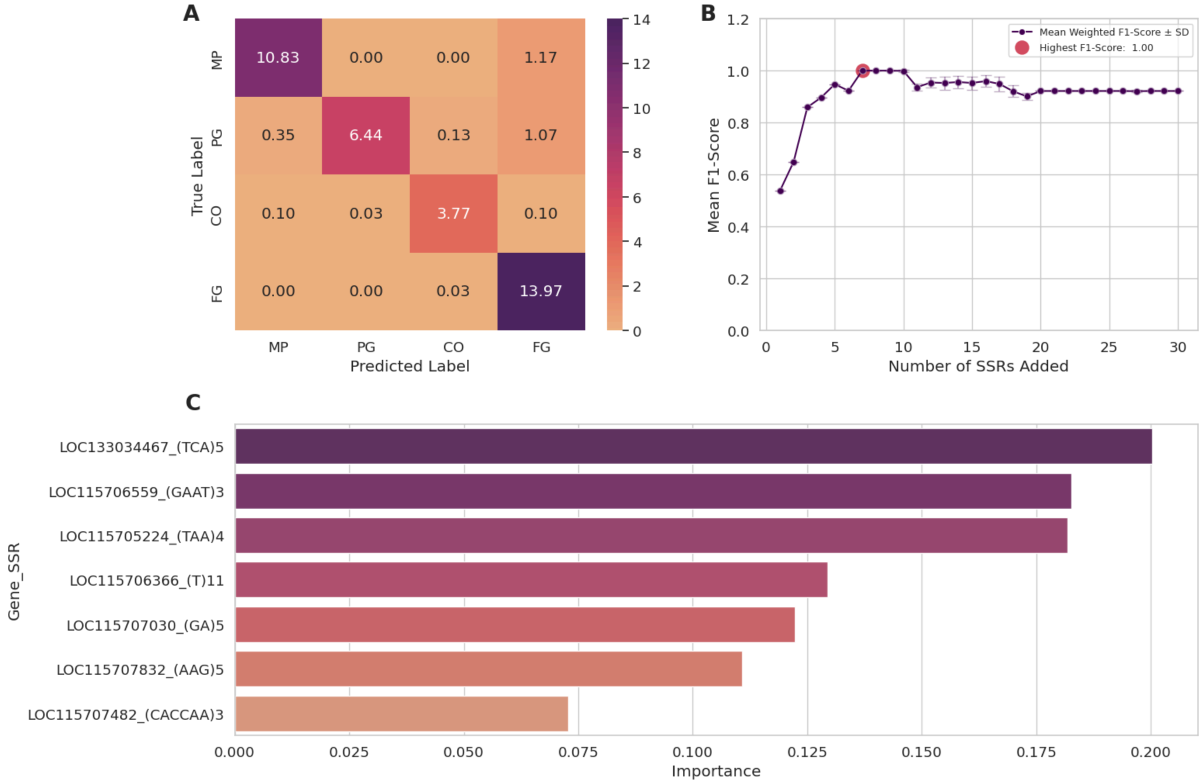

Figure S4. Evaluation of the GB Model using Subset 1 selected SSRs (GC-SSR FS). (A) Shows the average confusion matrix across all replicas, illustrating the classification performance across four sample origins: MP, PG, CO, and FG. (B) Weighted F1-score learning (with standard deviations) curve as a function of the number of SSR markers added. The red point marks the highest observed F1-score. (C) Feature importances of the 7 SSRs used in the GB model with highest score.

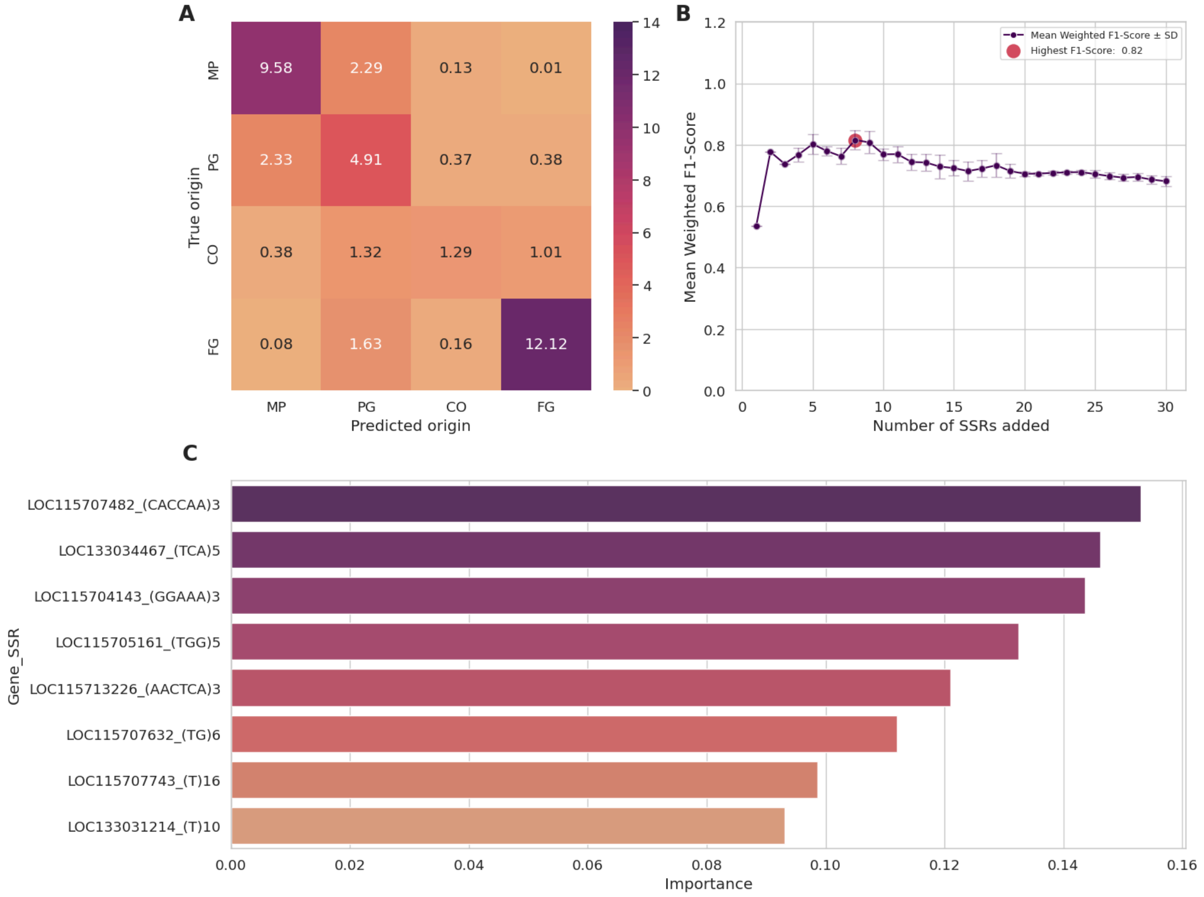

Figure S5. Evaluation of the GB Model using Subset 2 selected SSRs (SelectKBest FS). (A) Shows the average confusion matrix across all replicas, illustrating the classification performance across four sample origins: MP, PG, CO, and FG. (B) F1-score learning curve as a function of the number of SSR markers added. The curve depicts the mean weighted F1-scores (with standard deviations) across different feature sets. The red point marks the highest observed F1-score.(C) Feature importances of the 8 SSRs used for classification task in the GB model with highest score

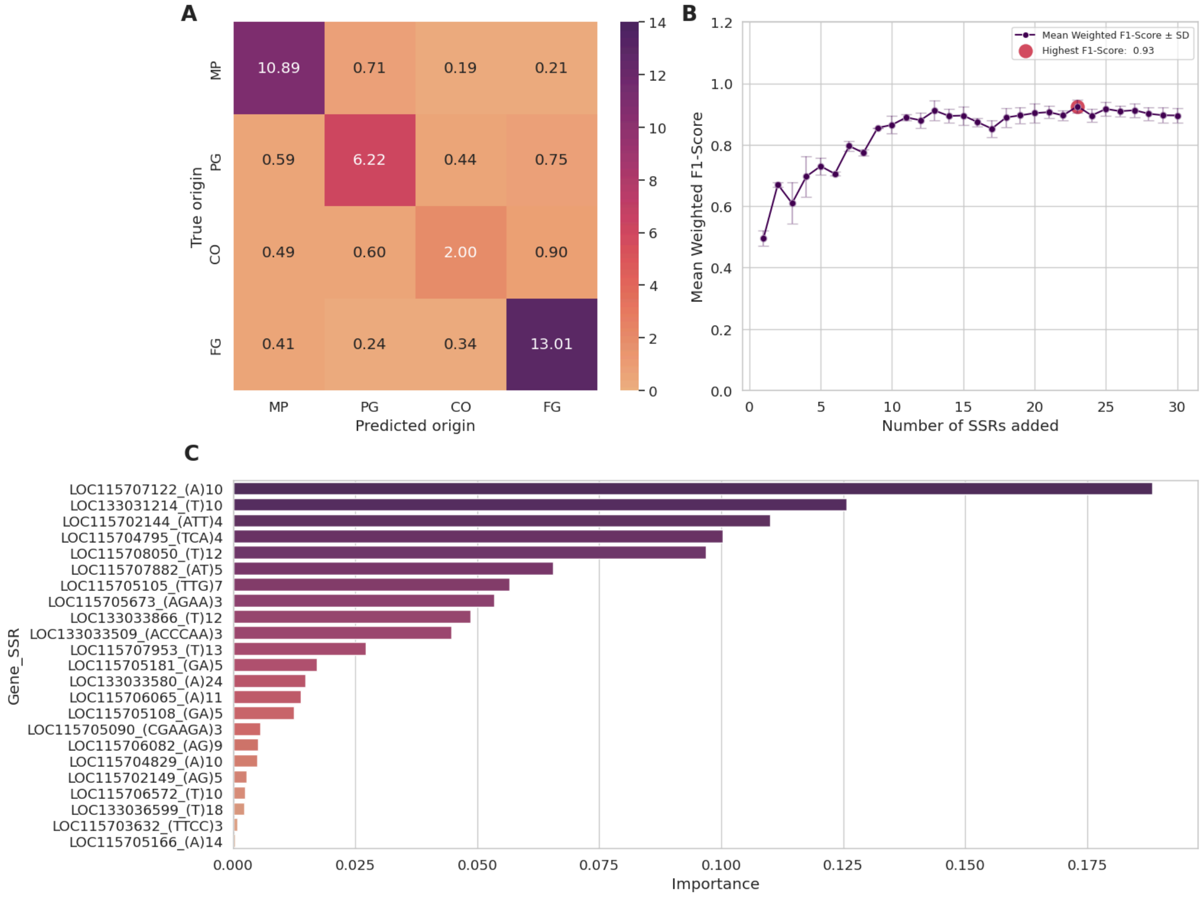

Figure S6. Evaluation of the GB Model using Subset 3 selected SSRs (Hybrid FS). (A) Shows the average confusion matrix across all replicas, illustrating the classification performance across four sample origins: MP, PG, CO, and FG. (B) F1-score learning curve as a function of the number of SSR markers added. The curve depicts the mean weighted F1-scores (with standard deviations) across different feature sets. The red point marks the highest observed F1-score.(C) Feature importances of the 23 SSRs used for classification task in the GB model with highest score

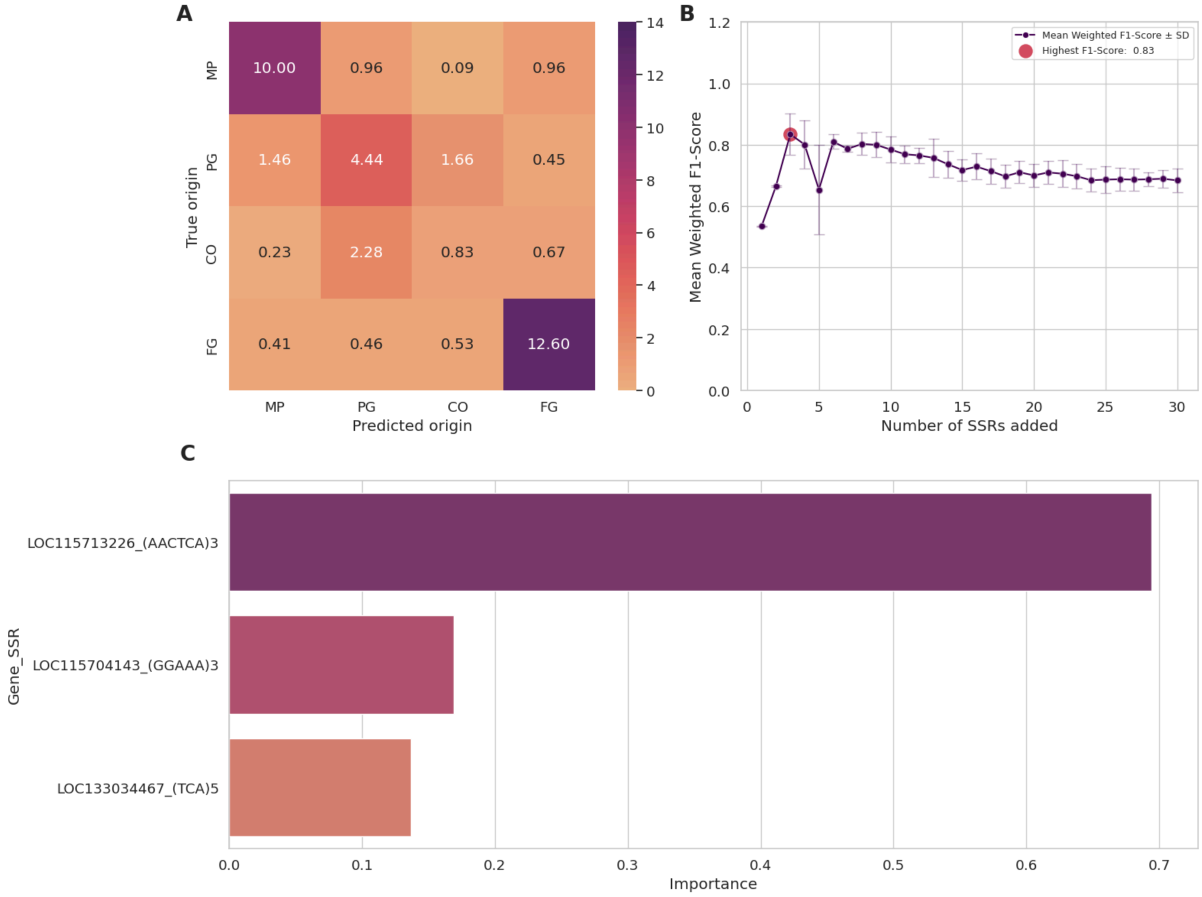

Figure S7. Evaluation of the GB Model using Subset 4 selected SSRs (LASSO FS). (A) Shows the average confusion matrix across all replicas, illustrating the classification performance across four sample origins: MP, PG, CO, and FG. (B) F1-score learning curve as a function of the number of SSR markers added. The curve depicts the mean weighted F1-scores (with standard deviations) across different feature sets. The red point marks the highest observed F1-score.(C) Feature importances of the 3 SSRs used for classification task in the GB model with highest score.

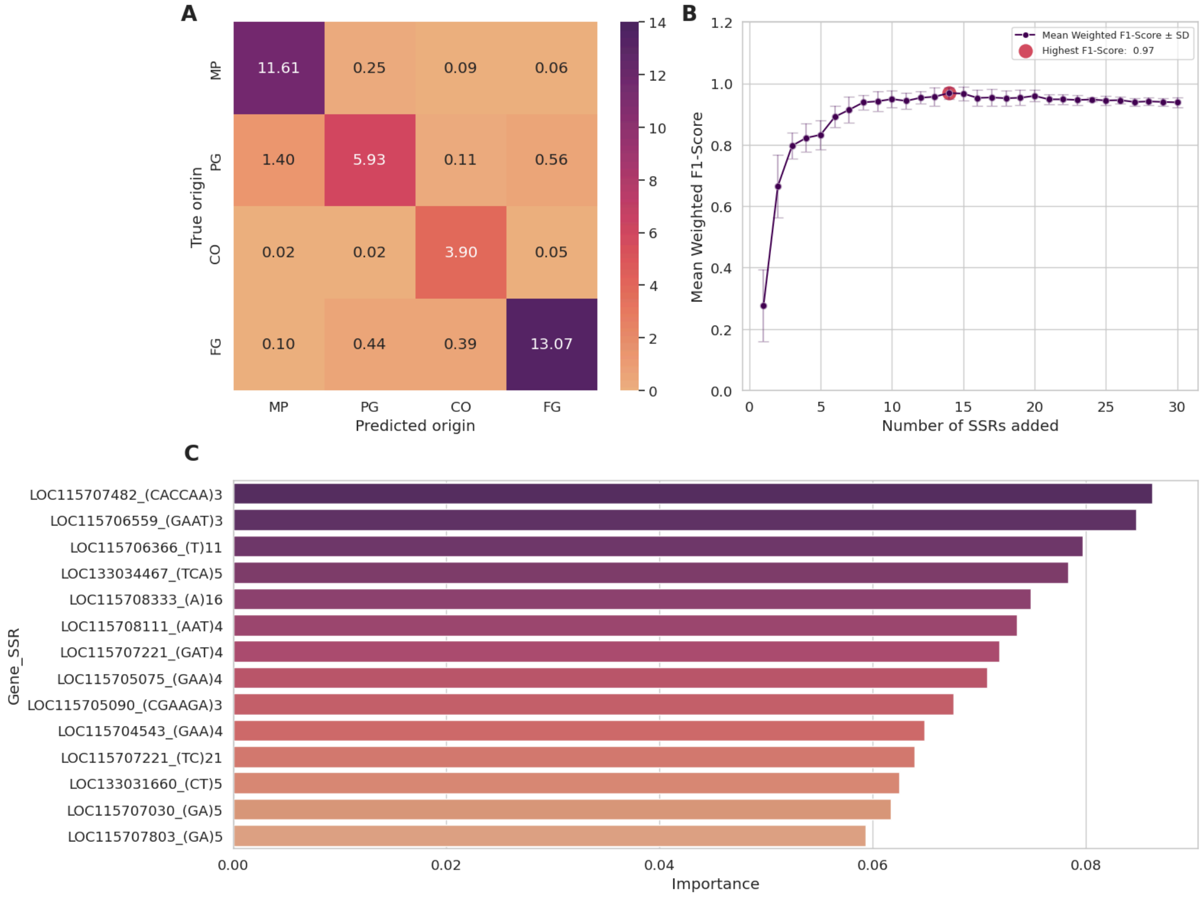

Figure S8. Evaluation of the RF classifier using Subset 1 selected SSRs (GC-SSR FS). (A) Shows the average confusion matrix across all replicas, illustrating the classification performance across four sample origins: MP, PG, CO, and FG. (B) F1-score learning curve as a function of the number of SSR markers added. The curve depicts the mean weighted F1-scores (with standard deviations) across different feature sets. The red point marks the highest observed F1-score.(C) Feature importances of the 14 SSRs used for classification task in the RF model with highest score.

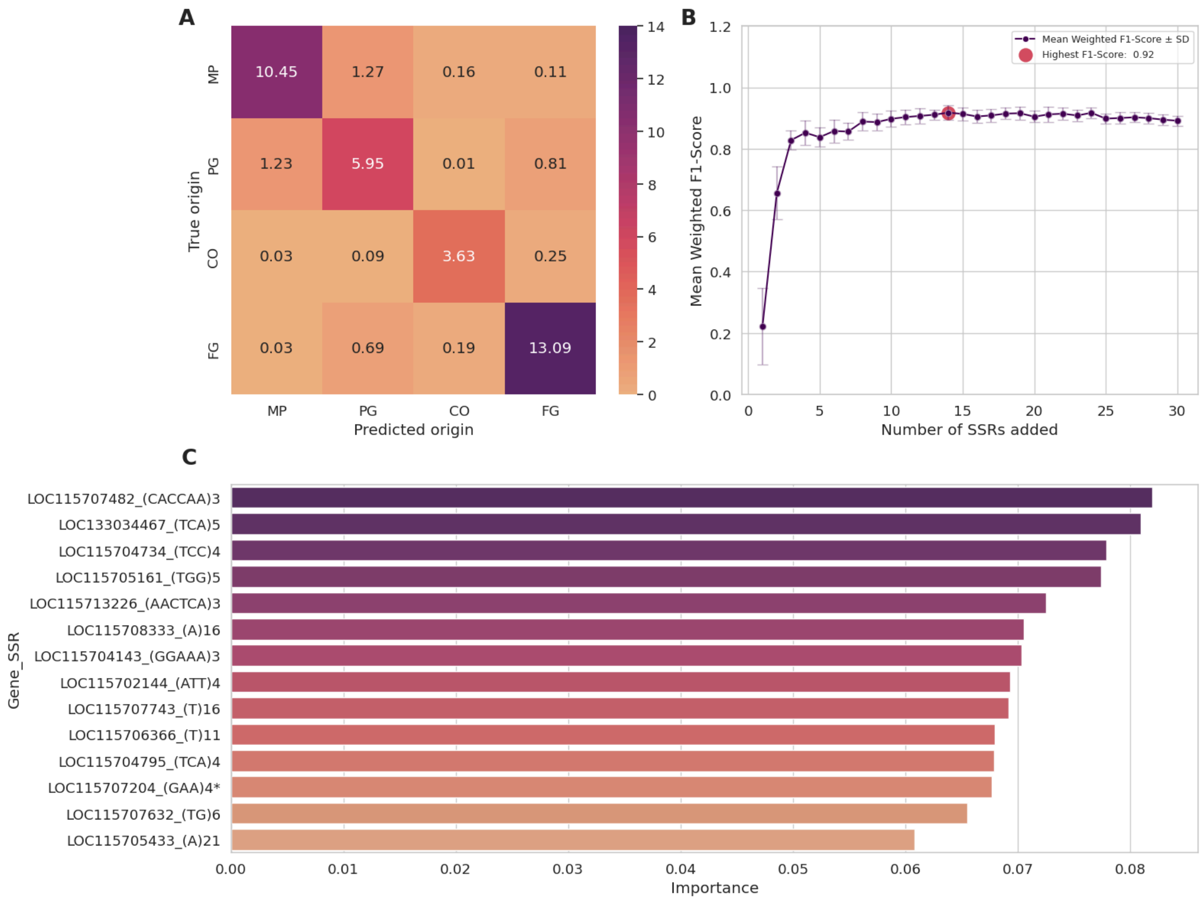

Figure S9. Evaluation of the RF classifier using Subset 2 selected SSRs (SelectKBest FS). (A) Shows the average confusion matrix across all replicas, illustrating the classification performance across four sample origins: MP, PG, CO, and FG. (B) F1-score learning curve as a function of the number of SSR markers added. The curve depicts the mean weighted F1-scores (with standard deviations) across different feature sets. The red point marks the highest observed F1-score.(C) Feature importances of the 14 SSRs used for classification task in the RF model with highest score.

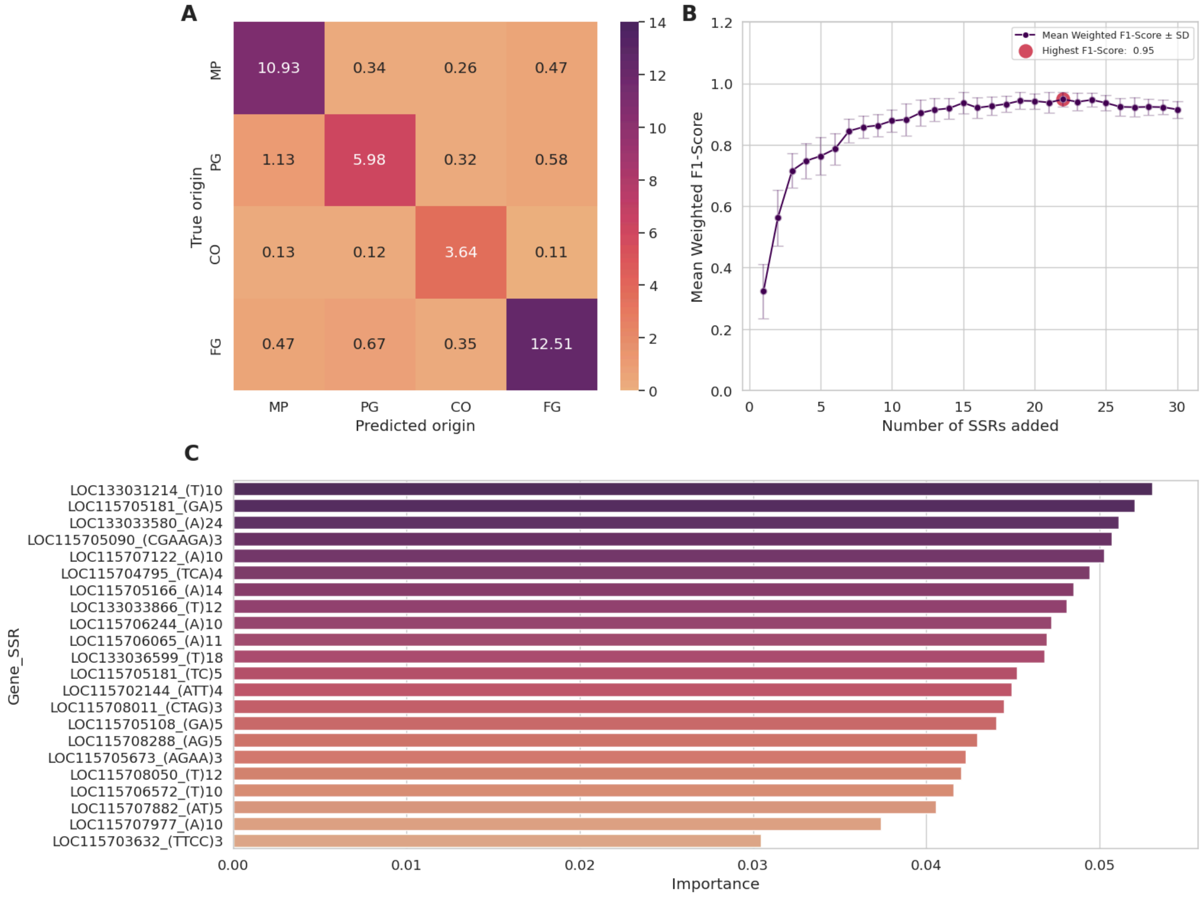

Figure S10. Evaluation of the RF classifier using Subset 3 selected SSRs (Hybrid FS). (A) Shows the average confusion matrix across all replicas, illustrating the classification performance across four sample origins: MP, PG, CO, and FG. (B) F1-score learning curve as a function of the number of SSR markers added. The curve depicts the mean weighted F1-scores (with standard deviations) across different feature sets. The red point marks the highest observed F1-score.(C) Feature importances of the 22 SSRs used for classification task in the RF model with highest score.

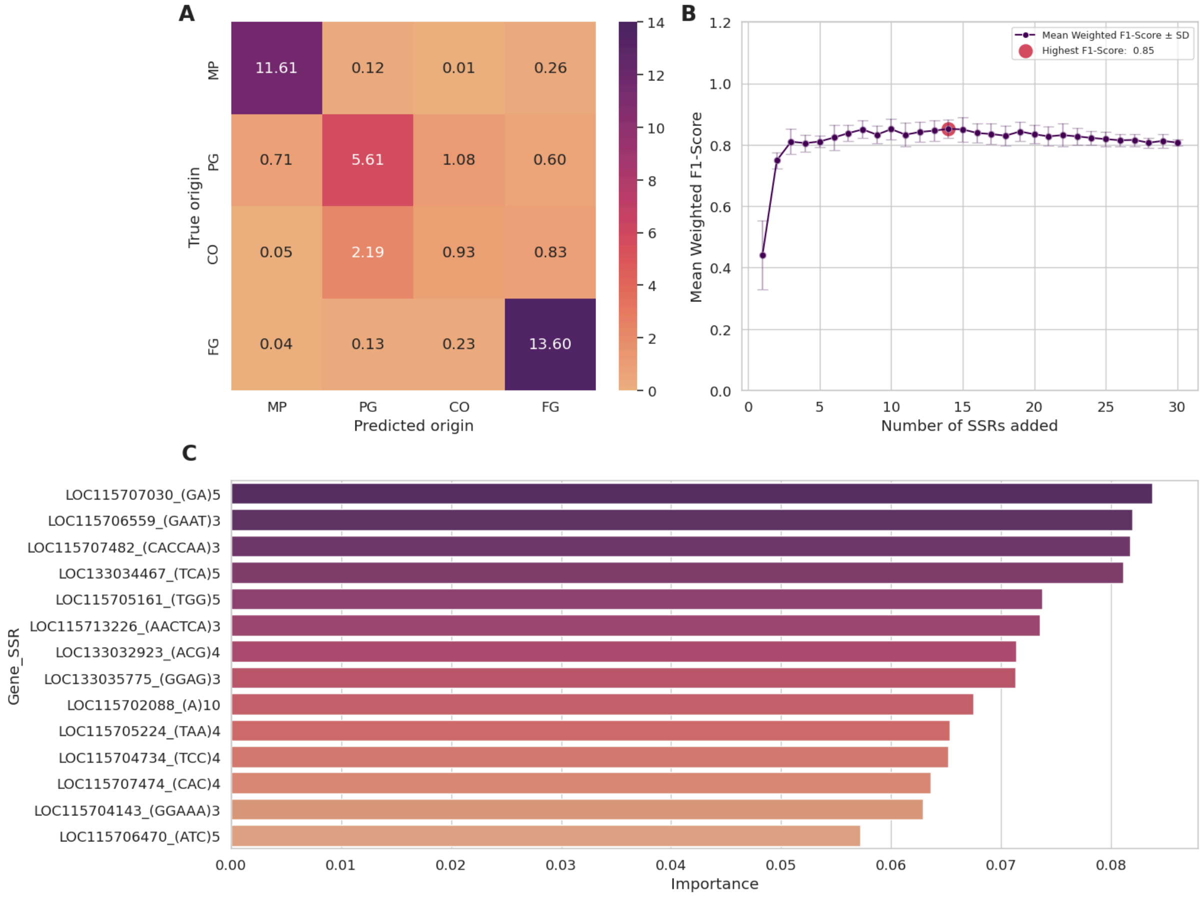

Figure S11. Evaluation of the RF classifier using Subset 4 selected SSRs (LASSO FS). (A) Shows the average confusion matrix across all replicas, illustrating the classification performance across four sample origins: MP, PG, CO, and FG. (B) F1-score learning curve as a function of the number of SSR markers added. The curve depicts the mean weighted F1-scores (with standard deviations) across different feature sets. The red point marks the highest observed F1-score.(C) Feature importances of the 14 SSRs used for classification task in the RF model with highest score.

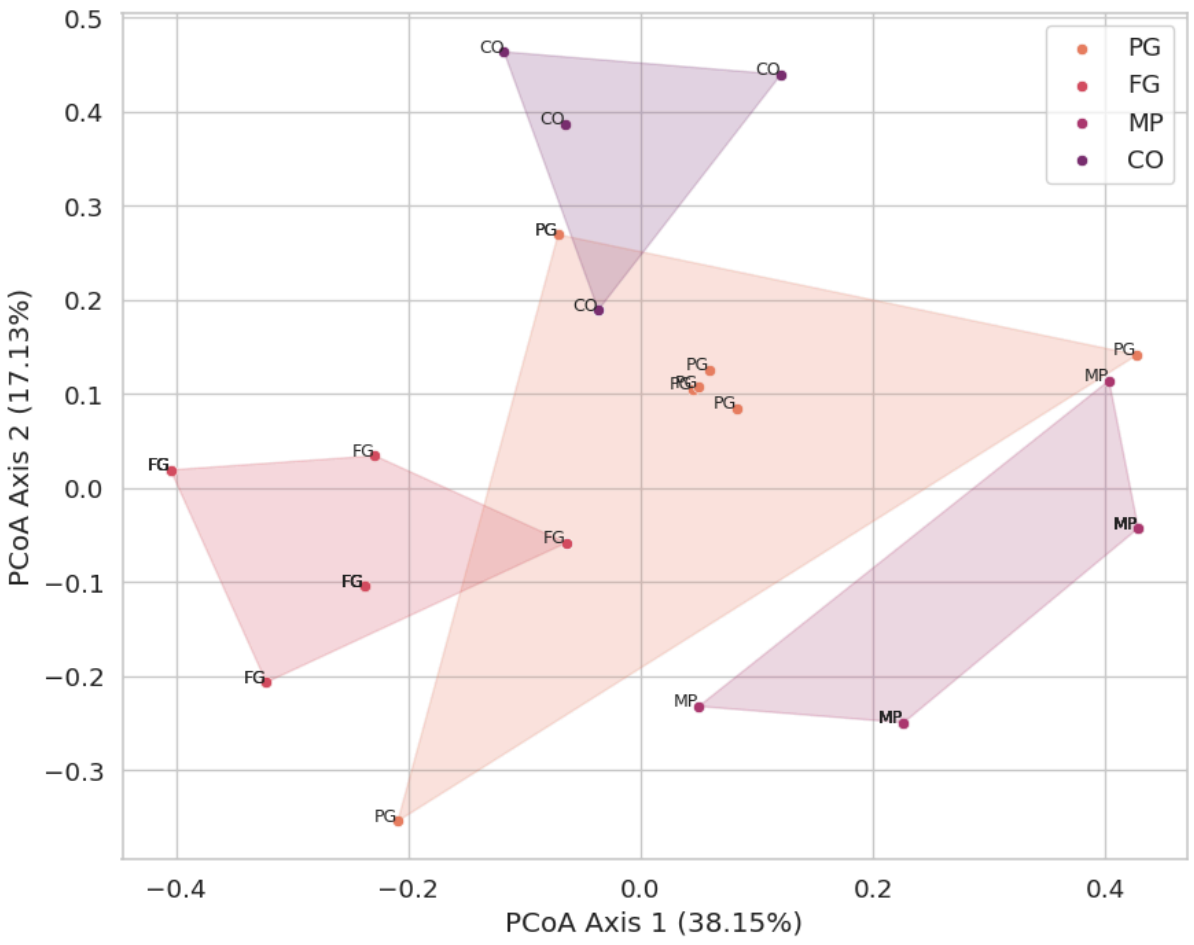

Figure S12: Principal Coordinate Analysis (PCoA) plot based on Jaccard distance calculated from the 7 SSR markers used in GB best model. The plot shows the ordination of samples across two dimensions (PC1 and PC2), which together explain 38.15% and 17.13% of the variation, respectively.

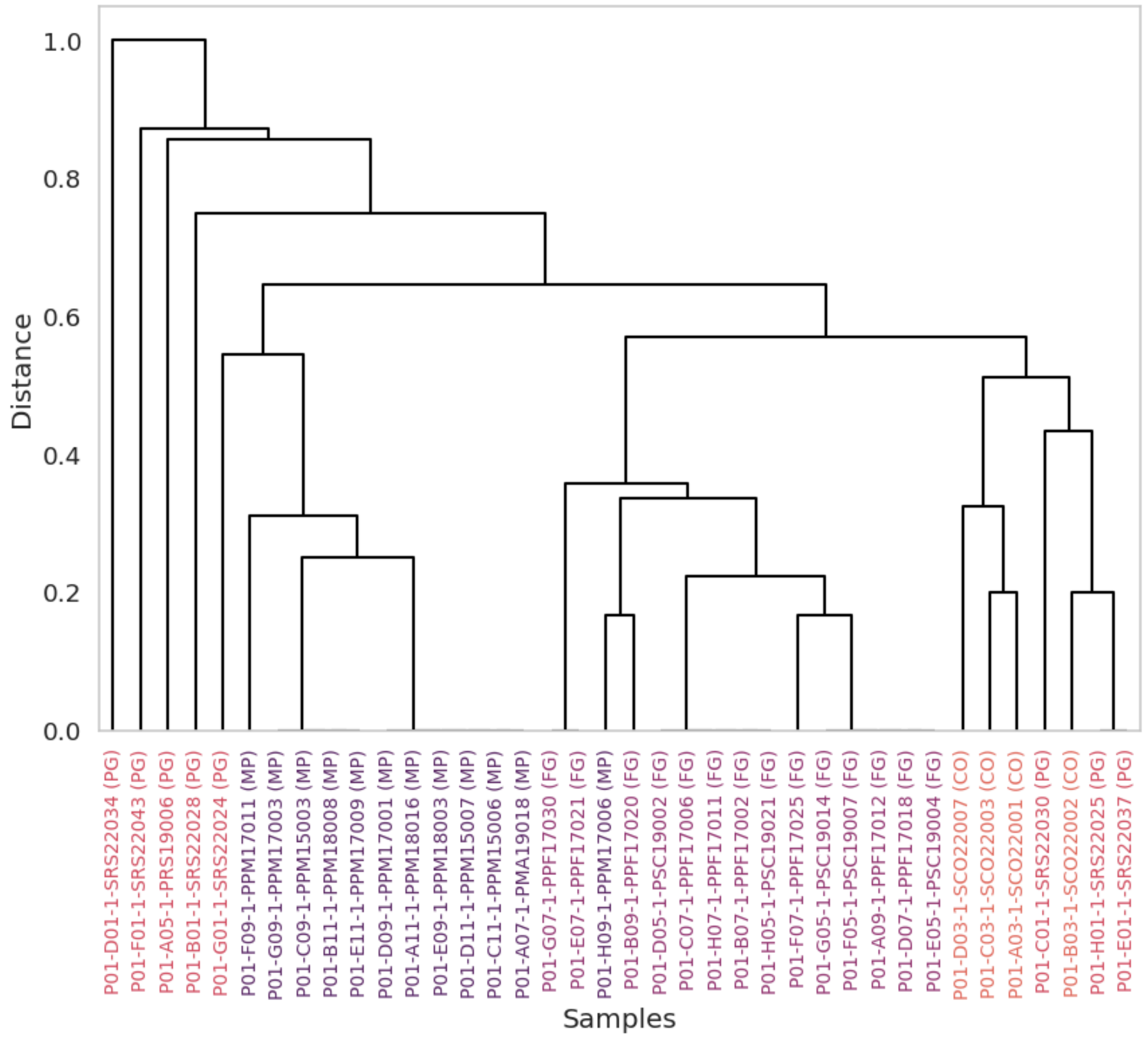

Figure S13: Hierarchical clustering dendrogram based on UPGMA method and Jaccard distance. The dendrogram illustrates the genetic relationships between samples using the 7 SSR markers (Subset 1) used in the GB best model. The samples are color-coded according to their origin, with MP in purple, PG in red, CO in orange-red, and FG in darker purple.

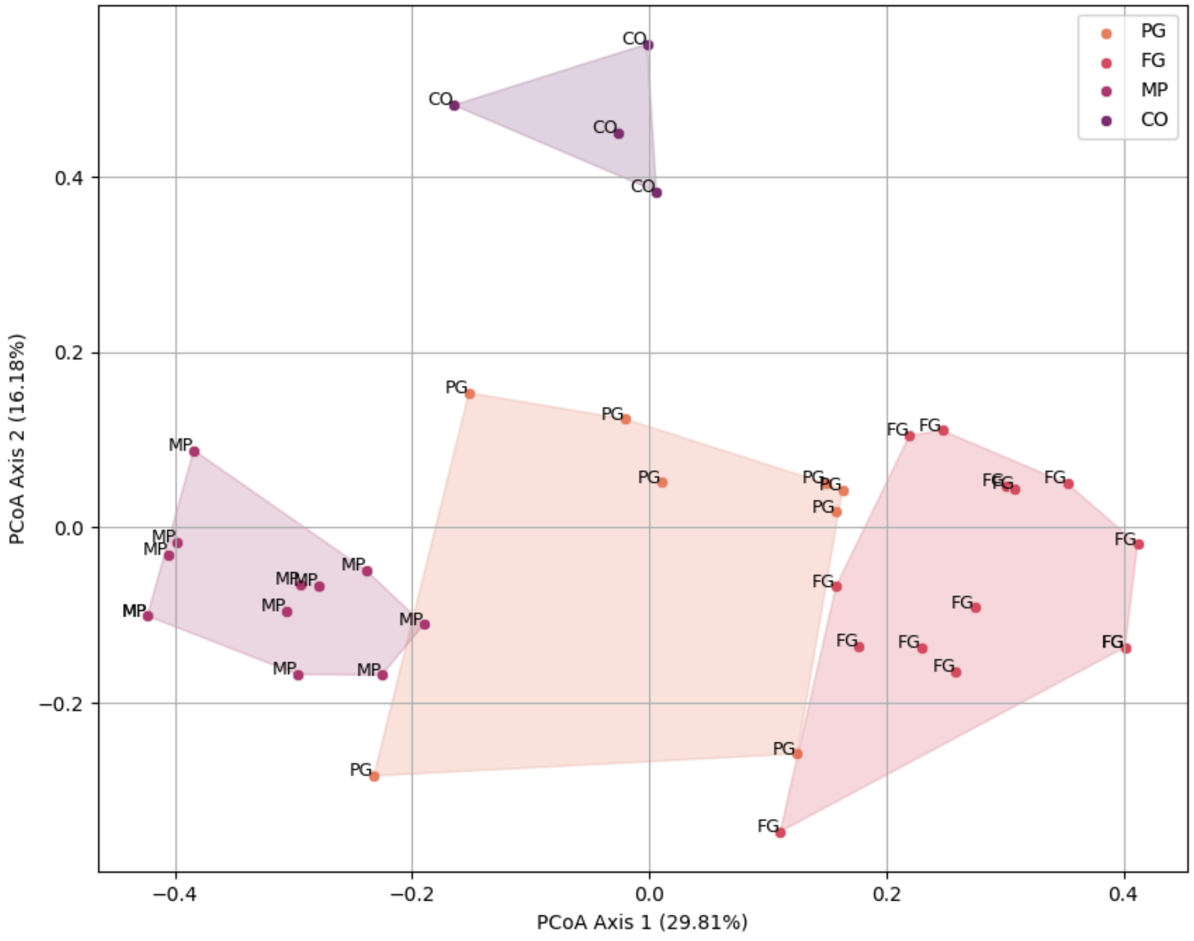

Figure S14: Principal Coordinate Analysis (PCoA) plot based on Jaccard distance calculated from the 14 SSR markers used in RF best model. The plot shows the ordination of samples across two dimensions (PC1 and PC2), which together explain 29.81% and 16.18% of the variation, respectively.

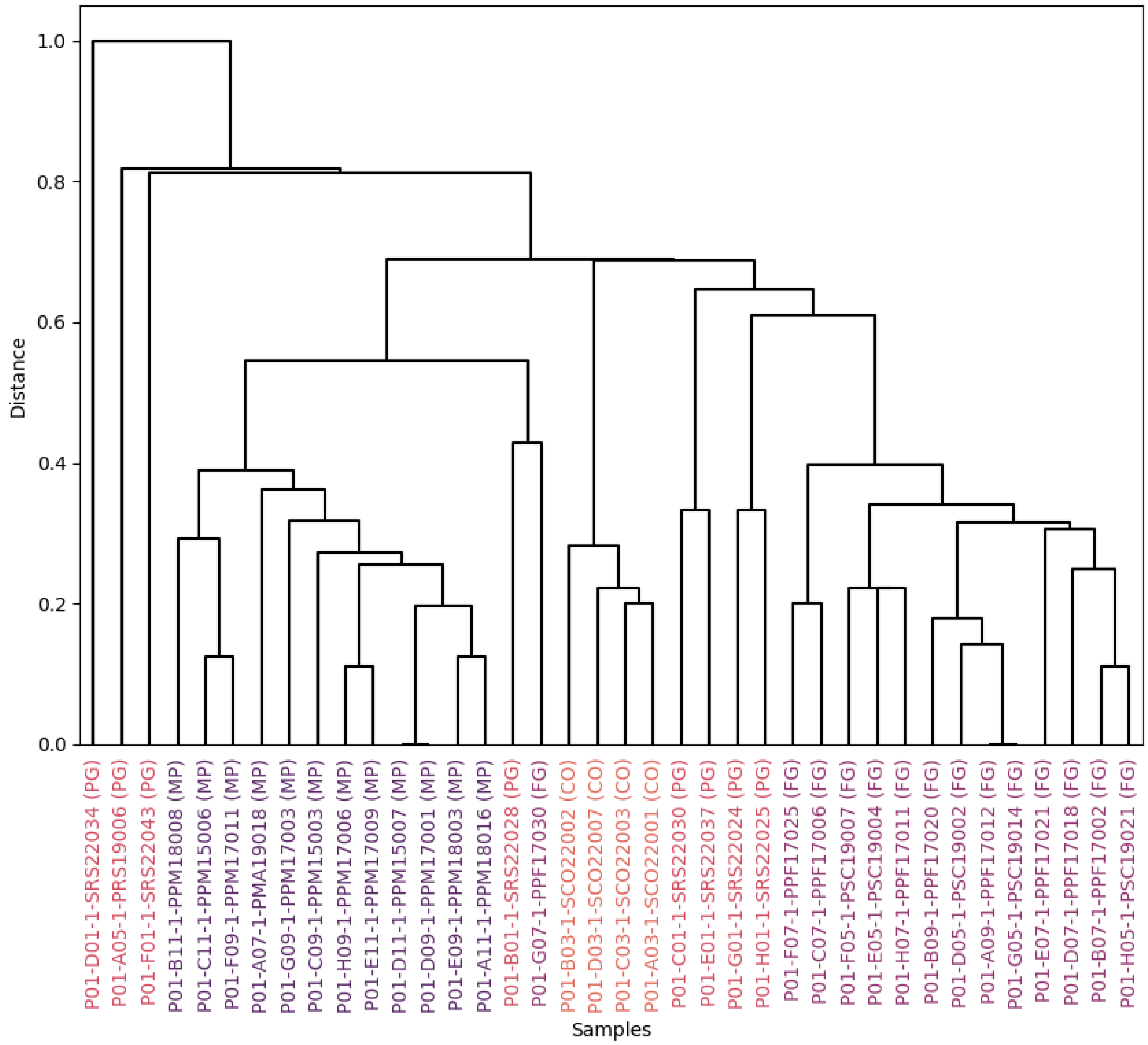

Figure S15: Hierarchical clustering dendrogram based on UPGMA method and Jaccard distance. The dendrogram illustrates the genetic relationships between samples using the 14 SSR markers (Subset 1) used in RF best model. The samples are color-coded according to their origin, with MP in purple, PG in red, CO in orange-red, and FG in darker purple.
